# Evaluation of rotavirus, pneumococcal conjugate and human papillomavirus vaccination in four Pacific island countries: A cost-effectiveness modelling study

**DOI:** 10.1371/journal.pmed.1004604

**Published:** 2026-02-12

**Authors:** Natalie Carvalho, Emma Watts, Victoria L. Oliver, Andrew Clark, Murat Hakan Ozturk, Siale Akauola, Clare Whelan, Take Naseri, Kylie Jenkins, Inez Mikkelsen-Lopez, Ki Fung Kelvin Lam, Rommel Rabanal, Ross McLeod, Mark Jit, Fiona M. Russell

**Affiliations:** 1 Melbourne School of Population and Global Health, University of Melbourne, Melbourne, Australia; 2 Asia-Pacific Health, Murdoch Children’s Research Institute, Melbourne, Australia; 3 London School of Hygiene and Tropical Medicine, London, United Kingdom; 4 UNICEF Pacific Islands Office, Suva, Fiji; 5 Ministry of Health, Tofoa, Tonga; 6 Ministry of Health, Funafuti, Tuvalu; 7 Ministry of Health, Apia, Samoa; 8 Telethon Kids, Perth, Australia; 9 Asian Development Bank, Mandaluyong City, Philippines; 10 eSYS Development, Sydney, Australia; 11 School of Global Public Health, New York University, New York, New York, United States of America; 12 Department of Paediatrics, The University of Melbourne, Melbourne, Australia; University of Bern Faculty of Natural Sciences: Universitat Bern Philosophisch-naturwissenschaftliche Fakultat, SWITZERLAND

## Abstract

**Background:**

The introduction of rotavirus vaccine (RVV), pneumococcal conjugate vaccine (PCV) and human papillomavirus vaccine (HPVV) has been slow in Pacific Island Countries, particularly among middle-income countries. To assist decision-making on the simultaneous introduction of these three vaccines, cost-effectiveness and budget impact evaluations were undertaken in Samoa, Tonga, Tuvalu and Vanuatu, using locally relevant data.

**Methods and findings:**

A proportionate outcomes model was used to evaluate vaccine introduction in each country from a health systems perspective, using country-specific data supplemented with regional and global estimates. A 10-year vaccination program was modelled from 2021, with costs and outcomes (disability-adjusted life years [DALYs]) summed over a life-time horizon and discounted at 3%. Vaccine dose costs were based on Pan American Health Organization (PAHO) Revolving Fund prices, with lower-priced products also explored. Introduction of all three vaccines in all countries could prevent over 1,000 deaths over the lifetimes of the vaccinated cohorts. The cost per DALY averted at PAHO Revolving Fund prices ranged from 42% to 73% of the per capita gross domestic product (GDP) in each country, and 15% to 58% for lower-priced vaccines. The budget impact ranged from 359% (Samoa) to 1,368% (Vanuatu) of the 2019 vaccine budgets, and 149% (Samoa) to 775% (Vanuatu) for lower-priced vaccines. Cost-effectiveness results were most sensitive to disease burden, discount rate, vaccine efficacy, and program costs. A limitation of our study is the reliance on data from Fiji to inform disease burden, as availability of country-specific data was limited.

**Conclusions:**

With development partner support, introduction of HPVV, PCV and RVV may represent good value for money in Samoa, Tonga, Tuvalu and Vanuatu, depending on willingness to pay thresholds. However, inclusion of these three vaccines will place considerable burden on immunisation budgets. Financial sustainability requires increases in immunisation budgets and negotiation of affordable vaccine prices. This analysis provides evidence of the benefit of introducing new vaccines, but shows the importance of affordable pricing to ensure sustainability for small Pacific Island countries.

## Author summary

**Why was this study done?:**

- Effective vaccines against pneumococcus, rotavirus and human papillomavirus have been available on the market for over a decade but are yet to be introduced into routine immunisation programs in some Pacific Island countries, with substantial consequences for population health.
- This study was commissioned by policy-makers from Samoa, Tonga, Tuvalu and Vanuatu and development partners to provide evidence to support decisions on whether to introduce three new vaccines into the immunisation schedule.

**What did the researchers do and find?:**

- We analysed the costs and health impacts of simultaneous introduction of pneumococcal conjugate vaccine, rotavirus vaccine and human papillomavirus vaccine in Samoa, Tonga, Tuvalu and Vanuatu, with development partner assistance.
- We show that introducing all three vaccines (at prices currently accessible to these countries) is good value for money in all four countries based on thresholds used by decision-makers at the time the program was adopted.
- Access to cheaper vaccines would be necessary for this program to be good value for money according to more recent thresholds which consider constraints in health budgets.
- The estimated annual costs of the vaccination program represented 359% (Samoa) to 1,368% (Vanuatu) of the 2019 vaccine budgets.

**What do these findings mean?:**

- Introduction of these vaccines may be a cost-effective way to improve population health in Pacific-Island countries, depending on vaccine pricing and thresholds used to determine value for money.
- We demonstrate that substantial increases in the immunisation budget are required to finance these vaccines, particularly in the longer term with development partner assistance scaling down over time.
- These findings provide evidence for the benefit of introducing new vaccines, but show the importance of affordable pricing to ensure sustainability for small Pacific Island countries.
- A limitation of our study is that much of the input into our model was data from Fiji because country-specific data that could be used for analysis were scarce.

## Abstract 3

In a modelling study, Natalie Carvalho and colleagues assessed the cost-effectiveness of simultaneously introducing rotavirus, pneumococcal conjugate and human papillomavirus vaccination in the Pacific Island countries Samoa, Tonga, Tuvalu and Vanuatu.

## Introduction

Effective vaccines against common causes of childhood and cervical cancer mortality have existed for 10–20 years and have formed part of comprehensive immunisation programs globally. The World Health Organization (WHO) recommends all infants be routinely immunised with rotavirus vaccine (RVV) to protect against rotavirus disease [1], which causes over a third of diarrhoea-related mortality [2]. Pneumococcal conjugate vaccines (PCVs) are also recommended by WHO to be administered to all children for the prevention of pneumococcal disease, a leading cause of lower respiratory tract infections in children [3]. A vaccine against human papillomavirus vaccine (HPVV), the predominant cause of cervical cancer, is recommended for administration to girls aged nine years and over as part of WHO’s Cervical Cancer Global Elimination Strategy [4]. All three vaccines are part of the immunisation programs in many countries in the Asia Pacific region, including Australia, New Zealand, Fiji, Indonesia and the Philippines. However, at the time of this analysis these vaccines were yet to be introduced in nine Pacific Island countries [5]. In 2012, Fiji was the first country to successfully adopt all three vaccines together. Evidence from both Fiji and Kiribati shows a decline in morbidity and mortality due to severe diarrhoea, a decrease in childhood pneumonia hospital admissions in Fiji following vaccine introduction, and high effectiveness of HPVV against HPV detection in Fiji [6–9].

Many Pacific Island countries are characterised by having small and highly dispersed populations, together with limited resources, remoteness, susceptibility to natural disasters and other external shocks, which increase dependence on external support for financing of immunisation programs (see S1 Text for further detail). Most Pacific middle-income countries are ineligible for Gavi support and, due to small population sizes, lack the bargaining power to negotiate affordable vaccine prices on their own. However, the UNICEF Vaccine Independence Initiative (VII) has been serving Pacific Island Countries with demand consolidation and bridge financing support [10]. In 2018, a unique window of opportunity arose when development partner assistance through the Asian Development Bank (ADB) became available to fund vaccine introductions in Samoa, Tonga, Tuvalu and Vanuatu through a five-year project and cost-sharing arrangement [11]. The specific vaccines selected for consideration of introduction were HPVV, RVV and PCV, which were chosen based on successful introduction in Fiji, the strong evidence base for disease reduction, and the need to prevent cervical cancer. Simultaneous introduction of all three vaccines was proposed due to the significant resource requirement underlying new vaccine introduction in these settings. The project leveraged the VII, enabling access to vaccine prices similar to those obtained by the Pan American Health Organization (PAHO) Revolving Fund for Access to Vaccines [12], but much more expensive than Gavi prices. In this study, we estimate the cost-effectiveness and budget impact of simultaneous roll-out of HPVV, RVV and PCV in Samoa, Tonga, Tuvalu and Vanuatu. Results from this analysis formed part of the package of evidence informing the decision to adopt all three vaccines in all four countries. Implementation of these vaccines in each country was delayed due to the COVID-19 pandemic, but began in 2021/2022.

## Methods

### Study design

We conducted a cost-effectiveness and budget impact analysis of introducing three new vaccines in four Pacific Island countries: Samoa, Tonga, Tuvalu and Vanuatu. Vaccines included: RVV, delivered to children under 5 by a two-dose schedule alongside diphtheria–tetanus–pertussis (DTP)-containing vaccine doses 1 (6 weeks) and 2 (10 weeks); PCV (PCV13), administered to children under 5 by a three-dose schedule alongside DTP-containing vaccine doses 1–3 (at 6, 10 and 14 weeks); and one dose of HPVV (bivalent HPV2 or quadrivalent HPV4), administered to ten-year-old girls alongside existing school-based vaccine programs. In a separate analysis, we also evaluated lower price vaccines for RVV (a three-dose schedule), PCV (three dose schedule of PCV10) and HPVV (one dose schedule of bivalent HPV2) that have more recently become available. There is insufficient evidence to support modelling of differential efficacy across the different brands of vaccines and where possible this analysis was designed to be brand-agnostic in line with WHO position papers and UNICEF policies supporting transparent and competitive procurement principles. We used input parameters (such as vaccine cost and efficacy) that were applicable to both PCV10 and PCV13 (given no clear difference in protection against pneumonia outcomes [13]); as well as HPV2 and HPV4 vaccines.

**Fig 1:**
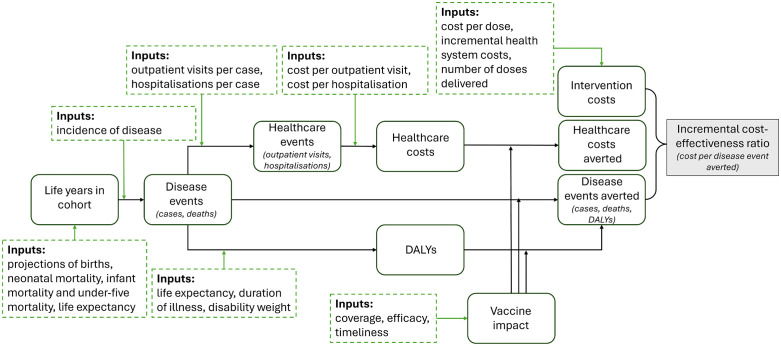
Simplified schematic of UNIVAC model structure. DALY, disability-adjusted life year.

A 10-year vaccination program (2021–2030) was modelled assuming simultaneous introduction of all three vaccines into routine immunisation programs (without a catch-up campaign), compared to no vaccine. We used UNIVAC (version 1.4), an Excel-based proportionate outcomes model that has been used extensively around the world to evaluate the cost-effectiveness of these three vaccines in low- and middle-income countries (LMICs) [14–18]. The model has been specifically designed for ease of use at country level [19], and it provides a basis for strengthening national capacity where feasible, building consensus between stakeholders and increasing the local ownership and policy-relevance of results. See Fig 1 and S2 Text for an overview of model structure and how inputs are combined to produce outputs. Briefly, estimates of costs (vaccine program costs and healthcare costs), health benefits (cases, disability and death) and cost-effectiveness are calculated by tracking the experience of annual birth cohorts to age five years for PCV and RVV, and annual cohorts of girls aged 10 throughout a lifetime horizon for HPVV. Each vaccine was modelled separately in UNIVAC (compared to the status quo) and results were combined to provide estimates of the joint vaccine introduction scenario.

We used a healthcare payer perspective in the base case, including both government and development partner costs, and considered a partial societal perspective that included household costs in a separate scenario. All costs were adjusted to 2019 United States dollars (USD) to reflect the year of decision-making. Costs in other currencies were first exchanged to local currency units (LCU) using World Bank year-specific exchange rates. Costs in LCU were inflated to 2019 LCU using country-specific World Bank gross domestic product (GDP) deflators, then exchanged to 2019 USD using World Bank USD/LCU 2019 exchange rates [20].

Where available, country-specific data on disease burden were used as model inputs. However, due to a scarcity of local data, data were predominantly sourced from published studies undertaken in other Pacific Island countries (particularly Fiji) or informed by global estimates. Rotavirus and pneumococcal disease events (cases, outpatient visits, hospitalisations and deaths) and treatment costs were estimated for successive birth cohorts over the first five years of life while cervical cancer events (cases, hospitalisations, and deaths) and treatment costs were estimated over the entire lifetime of a woman, with and without each vaccine. Average life expectancy, duration of illness and disability weights were used to estimate disability-adjusted life years (DALYs) over a life-time perspective, with future costs and benefits discounted at 3% in the base case.

This study is reported as per the Consolidated Health Economic Evaluation Reporting Standards 2022 (CHEERS 2022) Statement (S1 Checklist).

### Disease burden

The model multiplies life-years between birth and age five years by rates of disease cases and deaths (per 100,000) to estimate numbers of cases and deaths expected to occur without vaccination for PCV and RVV. For HPV, rates of cervical cancer (per 100,000) are applied to the number of females expected to be alive in each 5-year age group over the lifetime of the cohort. Inputs used for modelling disease burden are presented in Table 1, with further details provided in S1 Appendix.

**Table 1:** Inputs for modelling disease burden.

	Base case values (low estimate, high estimate)				Source/Notes
	Samoa	Tonga	Tuvalu	Vanuatu	
**Cervical Cancer**					
Cases (per 100,000 per year)	Age-specific incidence. See appendix.				
Hospitalisations (% of cases hospitalised)					
Local or regional cases	2.5%	1.1%	1.7%	5.7%	Based on opportunistic screening rates^a^
Distant cases	100%	100%	100%	100%	Assumption
Deaths (per 100,000 per year)	Age-specific incidence. See appendix.				
**Pneumococcal disease** ^b^					
Cases (per 100,000 per year, 0–5 years)					
Acute otitis media	1,900 (950, 11,555)	1,900 (950, 11,555)	1,900 (950, 11,555)	1,900 (950, 11,555)	Base case/low: see note^c^; High: see note^d^
Pneumonia (non-severe)	1,122 (968, 1,333)	1,095 (945, 1,302)	936 (807, 113)	1,707 (1,473, 2,029)	Base case: median estimates from [21]; Low/high: see note^e^
Pneumonia (severe)	435 (326, 496)	425 (319, 485)	361 (270, 411)	667 (500, 761)	Base case: median estimates from [21]; Low/high: see note^e^
Meningitis	19 (9.9, 28)	19 (9.9, 42)	19 (9.9, 47)	19 (9.9, 29)	Base case: point estimate from [22] see note^f^; Low: point estimate from [23]; High: see note^e^
NPNM	19 (9.9, 32)	19 (9.9, 47)	19 (9.9, 53)	19 (9.9, 33)	Base case/low: assume 1 case per Sp meningitis case; High: see note^e^
Meningitis sequelae	3.4 (1.8, 5.04)	3.4 (1.8, 7.5)	3.4 (1.8, 8.46)	3.4 (1.8, 5.22)	Assume 18% of bacterial meningitis had sequelae [23].
Visits (% of cases with outpatient visit)					
Acute otitis media	50%	50%	50%	50%	Assumption
Pneumonia (non-severe)	78%	64%	60%	72%	DHS data proxy condition: acute respiratory infection (Samoa and Vanuatu), fever (Tonga) or diarrhoea (Tuvalu)
Pneumonia (severe), meningitis, NPNM, meningitis sequelae	100%	100%	100%	100%	Assumption
Hospitalisations (% of cases hospitalised))					
Pneumonia (severe)	80%	90%	90%	80%	Assumption^g^
Meningitis, NPNM	100%	100%	100%	100%	Assumption
Deaths (per 100,000 per year 0–5 years)					
Pneumonia (severe)	9.9 (7, 10)	13 (9, 14)	21 (15, 22)	25.3 (18, 26.4)	Base case: median estimates from [21]; Low/high: see note^e^
Meningitis	7.6 (2, 11.2)	7.6 (3, 16.8)	7.6 (3, 18.8)	7.6 (2, 11.6)	Base case/high: case fatality ratio from [22] applied to base case and high incidence. Low: median incidence from [21]
NPNM	4.2 (2, 7.04)	4.2 (3, 10.34)	4.2 (3, 11.66)	4.2 (2, 7.26)	Base case/high: case fatality ratio from [24] applied to base case and high incidence.; Low: median incidence from [21]
**Rotavirus** ^b^					
Cases (per 100,000 per year, 0–5 years)					
Non-severe RVGE	9,685 (6,685, 13,542)	9,542 (6,685, 13,542)	9,685 (6,685, 13,542)	9,685 (6,685, 13,542)	See note^h^
Severe RVGE	315 (315, 458)	458 (315,458)	315 (315,458)	315 (315,458)	See note^i^
Intussusception	13.26	13.26	13.26	13.26	See note^j^
Visits (% of cases with outpatient visit)					
Non-severe RVGE	78%	64%	60%	72%	DHS data proxy condition: acute respiratory infection (Samoa and Vanuatu), fever (Tonga) or diarrhoea (Tuvalu)
Severe RVGE	100%	100%	100%	100%	Assumption
Hospitalisations (% of cases hospitalised)					
Severe RVGE, intussusception	100%	100%	100%	100%	Assumption
Deaths (case fatality risk, %)					
Severe RVGE	2.6%	2.6%	2.6%	2.6%	See note^k^
Intussusception	0.03 (0.02, 0.12)	0.03 (0.02, 0.12)	0.03 (0.02, 0.12)	0.03 (0.02, 0.12)	Base case/low/high: median, lower and upper uncertainty range from [25]

^a^Calculated as: number of pap smears conducted in 10-year period (based on most recent local data available scaled up to 10 years) divided by the number of women aged 20 years and up (UN Pop data) to get proportion of women who would get at least 1 pap smear in 10 years. This was multiplied by 55% pap smear sensitivity to get proportion of cases that would be diagnosed and hospitalised. In Samoa, account for data available of 18% unsatisfactory pap smear results. In Tuvalu, assume 10% of pap smears are repeats due to up to 1 year delay for results to be available (samples sent to Fiji).

^b^Under 5 incidence rates are multiplied by age distributions by week of age to determine the number of disease events expected to occur between birth and age 5 years.

^c^For the base case, a very conservative estimate of AOM incidence was made from the prevalence of AOM reported in a study of pacific islander children living in New Zealand [26]. The low estimate assumes 50% incidence of the base case.

^d^Calculating the global estimate for AOM incidence in children <5 years from Monasta and colleagues [27], we conservatively assumed 20% of cases are caused by Streptococcus Pneumoniae for the high input.

^e^Upper and/or lower estimates were from modelled upper and lower uncertainty estimates in Wahl and colleagues, where uncertainty intervals were based on jack-knife, leave-one-study-out approaches as described in [24].

^f^Assume 38% of invasive pneumococcal disease is meningitis.

^g^Assumption based on underlying child mortality in country and geographic access to hospitals.

^h^Estimated incidence of all severity rotavirus disease reported in Bilcke and colleagues [28] is 0.24 per child year in children under 2 years. We crudely assumed all cases in children under 5 years to occur in infants under 2 years to derive an incidence of 10,000 per 100,000 children under 5 per year ([0.24 + 0.24 + 0 + 0 + 0]/5 = 0.1). Incidence of severe disease (see note below) was subtracted from this to give incidence of non-severe disease.

^i^For Tonga the base case is derived from Tonga data collected during the project design: Estimated average annual incidence of hospitalised diarrhoea in U5 2012–2016 (incidence 1,175 per 100,000 per year) was multiplied by 0.39 presumed proportion caused by rotavirus, as documented in Fiji [29]. For Samoa, Tuvalu and Vanuatu, the base case is derived from Fiji published annual incidence of rotavirus in children under 5 [6]. For all countries, the low is the estimate from Fiji and the high is the estimate from Tonga.

^j^Published data from Fiji, adjusted to U5 assuming no hospitalisations >24 months.

^k^Case fatality risk of 2.6% from Fiji applied to base case, low, and high case incidence [29].

Abbreviations: DHS, demographic and health survey; NPNM, non-pneumonia non-meningitis invasive diseases; RVGE, rotavirus gastroenteritis.

#### Cervical cancer disease incidence and mortality.

Due to limited country-specific data at the time of this analysis, age-specific incidence and mortality rates of cervical cancer in all countries were based on published data from Melanesian Fijian women using actual cases rather than model estimates (Table B in S1 Appendix) [30]. These rates were mostly higher than more recent GLOBOCAN estimates that have become available for Samoa and Vanuatu [31], and higher than the most recent Global Burden of Disease (GBD) estimates [32]. We assumed a given distribution of incident cases across severity levels (19% local, 73% regional, and 9% distant incident cases), based on a recent global analysis that accounted for information about staging of cervical cancer cases in LMICs [33].

#### Pneumococcal disease incidence and mortality.

The model included six health conditions associated with pneumococcal disease including: acute otitis media, non-severe and severe pneumonia, meningitis, other non-pneumonia non-meningitis invasive diseases (NPNM) and meningitis sequelae. These health conditions have differing epidemiology and vaccine efficacy.

The incidence of acute otitis media across all countries was based on data on Pacific Island children living in New Zealand [34]. For the incidence of severe and non-severe pneumonia, we used country-specific estimates derived from WHO and Maternal and Child Epidemiology Estimation collaboration (MCEE) [21].

For the incidence of meningitis across all countries, we used data from a study of invasive pneumococcal disease in children under five in Tonga between 2010 and 2013, where 38% of cases were meningitis [22]. We assumed one case of non-pneumonia non-meningitis invasive disease per case of meningitis. We assumed 18% of bacterial meningitis cases were associated with sequelae based on a study of clinical manifestations of invasive pneumococcal disease in children under five in Fiji [23].

For the incidence of pneumococcal pneumonia deaths, we used country-specific estimates from WHO and MCEE [21]. A case fatality ratio of 40% for meningitis was from a published study of invasive pneumococcal disease in Tonga [22]. A case-fatality ratio of 22% for severe NPNM was based on a published estimate for the Western Pacific region [24]. The age distribution of pneumococcal disease events in each week of age under five years was based on a global review (Table C of S1 Appendix) [35].

#### Rotavirus disease incidence and mortality.

Cases of non-severe rotavirus gastroenteritis in children under five years old in each country were based on a global systematic review of symptomatic rotavirus infections in children [28]. For Tonga, the estimate for hospitalised rotavirus gastroenteritis was based on local data estimating the incidence of hospitalised gastroenteritis in children under five, assuming 39% of gastroenteritis hospital admissions were attributed to rotavirus as was found in Fiji [6]. For Samoa, Tuvalu and Vanuatu, the estimate was based on the Fiji study of incidence of hospitalised rotavirus gastroenteritis in children under five, in the years prior to vaccine introduction, as no local data were available [6]. We estimated that the incidence of severe rotavirus gastroenteritis was equal to the incidence of hospitalised cases. This resulted in a conservative assumption of the incidence of severe disease compared to that reported in vaccine trials [36].

Evidence of a case fatality ratio of 2.6% for hospitalised rotavirus gastroenteritis in children under five in Fiji was applied to the incidence of cases to estimate mortality in all countries [29]. The age distribution of rotavirus disease events in each week of age under five years was estimated from a global review and statistical analysis of 92 hospital admission datasets, stratified by child mortality strata [37].

#### Intussusception.

Rarely, RVV causes a serious adverse event, intussusception, which requires hospitalisation and treatment. The baseline incidence of intussusception was applied from a published study in Fiji, prior to RVV introduction [38] (Table C of S1 Appendix). We assumed a case fatality ratio of 0.03%, based on a meta-analysis for the Western Pacific region [25]. We estimated a relative risk of intussusception associated with RVV in the 1–7 day risk period based on a recent meta-analysis [39]. The age distribution of intussusception cases and deaths was based on a recent global review (Table C in S1 Appendix) [25].

#### Disability weights, duration and calculations of disability-adjusted life years.

To estimate years lived with disability, country-specific demographic data and life expectancy at birth were sourced from the United Nations Populations Projections (Table A in S1 Appendix). For non-fatal conditions, years lived with disability were based on disability weights multiplied by duration of illness. Disability weights for all vaccine-preventable diseases across all countries came from the GBD Study 2013 (Table D in S1 Appendix) [40].

The average duration of illness for local, regional and distant cervical cancer was based on the 2017 GBD study estimates [41]. For pneumococcal disease, we assumed an average duration of seven days for acute otitis media and non-severe pneumonia, and a duration of 10 days for severe pneumonia, meningitis and other severe, non-pneumonia non-meningitis disease. We assumed an average duration of 50 years for meningitis sequelae. For rotavirus, we used an average duration of three days for non-severe and seven days for severe gastroenteritis [42], and assumed a duration of seven days for intussusception.

Current and projected life expectancy estimates by age and year are used to calculate years of life lost due to premature mortality from the age of disease to death. Years of life with disease and years of life lost are summed to give DALYs attributed to the year of disease onset.

### Vaccine coverage, timeliness and efficacy

For PCV and RVV, estimates of vaccination impact are restricted to children aged under five years of age. The impact is calculated by multiplying the expected number of disease and healthcare events (cases, clinic visits, hospitalisations, deaths) in each week of age up to five years by the expected coverage of vaccination in each week of age (adjusted for realistic vaccine delays/timeliness) and the expected efficacy of vaccination in each week of age (adjusted for the waning vaccine protection).

For HPVV, vaccination impact is calculated for the target cohort (taking into account vaccine coverage, the cervical cancer type distribution, and the efficacy of the vaccine against each type), and is assumed to provide lifetime protection.

Inputs used to model vaccine coverage and efficacy are presented in Tables 2 and 3. In the status quo we model no coverage of PCV, RVV and HPVV. For the intervention, HPVV coverage assumptions were based on 2023 WHO/UNICEF Estimates of National Immunization Coverage (WUENIC) data on HPVV coverage in Fiji, where HPVV has been a part of the routine immunisation schedule for several years [53]. The model was updated using country-specific data on actual HPVV coverage in 2023 (following vaccine introduction) in the lower estimate for the sensitivity analysis. Routine coverage of RVV and PCV were based on country-specific coverage from WUENIC 2023 using DTP-containing vaccine doses 1–3 as proxy, given that these vaccines are given to infants together in a bundle.

**Table 2:** Vaccine coverage estimates (%).

	Base case values (low estimate, high estimate)			
	Samoa	Tonga	Tuvalu	Vanuatu
HPVV^a^				
Dose 1	87.0 (86.0, 87.0)	86.0 (30.0, 86.0)	86.0 (84.0, 86.0)	86.0 (41.0, 86.0)
PCV/RVV^b^				
Dose 1	100.0 (90.0, 100.0)	99.4 (89.5, 99.4)	100.0 (90, 100.0)	96.0 (86.4, 100.0)
Dose 2	91.9 (82.7, 91.9)	99.3 (89.4, 99.3)	97.0 (87.4, 97.0)	93.0 (83.7, 95.2)
Dose 3	83.2 (74.8, 83.2)	99.2 (89.3, 99.2)	94.1 (84.7, 94.1)	90 (81.0, 90.5)

^a^Except for Samoa, WUENIC data on coverage of first dose of HPVV among the target population (females) in 2023 in Fiji was used for the base case and high estimate. The low estimate used country-specific WUENIC data on coverage of first dose of HPVV among the target population (females) in 2023. In Samoa, country-specific data was used in the base case and high, while coverage data from Fiji was used as the low estimate.

^b^Samoa, Tonga and Tuvalu: Base case used official coverage estimates of DTP doses 1 and 3 (2023) from WHO/UNICEF and high used administrative coverage estimates (which in some cases were equivalent to official coverage estimates). For Vanuatu: due to a decline in immunisation coverage following the COVID-19 outbreak, data from 2019 were used. For all countries, low estimate was 90% of base case and dose 2 coverage was taken as a linear average of dose 1 and 3 coverage due lack of data to inform plausible estimates for these parameters.

Abbreviations: DTP, diphtheria–tetanus–pertussis; HPVV, human papillomavirus vaccine; PCV, pneumococcal conjugate vaccine; RVV, rotavirus vaccine; WUENIC, WHO/UNICEF Estimates of National Immunization Coverage.

**Table 3:** Vaccine efficacy and duration estimates. Vaccine efficacy from 2 weeks after vaccination unless otherwise stated. The same efficacy and duration estimates were used for all countries unless otherwise stated.

	Base case values (low estimate, high estimate)	Source/Notes
**HPVV**		
Dose 1 efficacy (%)	73.8 (59.0,79.0)	See note^a^
Duration of efficacy (years, all doses)	100	Assumption based on [43,44]
**PCV**		
Dose 1 efficacy (severe disease, %)^b^	29.0 (14.5, 37.5)	Assume dose 1 efficacy is 50% of dose 2
Dose 2/3 efficacy (severe disease, %)^b^		Base case/low/high: median, lower and upper uncertainty range from [45]
3 years after vaccination	58.0 (29.0, 75.0)	
4 years after vaccination	57.0 (28.5, 73.7)	
5 years after vaccination	28.2 (14.1, 36.5)	
Duration of efficacy (years, all doses)	5	Assumption based on [46]
**RVV (Samoa, Tonga, Tuvalu)**		
Dose 1 efficacy (severe disease, %)^c^	57.8 (45.5, 73.6)	See note^d^
Dose 2/3 efficacy (severe disease, %)^c^		
2 weeks after vaccination	91.4 (89.8, 92.7)	
6 months after vaccination	76.8 (72.7, 80.2)	
12 months after vaccination	69.5 (64.3, 73.9)	
**RVV (Vanuatu)**		
Dose 1 efficacy (severe disease, %)^c^	49.9 (38.2, 65.3)	See note^d^
Dose 2/3 efficacy (severe disease, %)^c^		Base/Low: see noted. High: see note^e^
2 weeks after vaccination	78.9 (75.5, 91.4)	
6 months after vaccination	45.7 (38.2, 76.8)	
12 months after vaccination	31.8 (23.8, 69.5)	

^a^Base case: Assume 97.5% efficacy against high-risk subtypes based on median efficacy of one dose in a study in Kenya [47] multiplied by percent of cancer caused by high-risk types, taken to be 75.7% based on median estimate for Oceania [48]. Low: Assume 81.7% efficacy against high-risk subtypes (HPV16/18) based on lower bound 95% confidence interval in Kenya study [47] multiplied by lower bound estimate for percent of cancer caused by high-risk types reported for Oceania (72.3%) [48]. High: Assume 100% efficacy against high-risk subtypes based on [49], multiplied by upper bound 95% confidence interval for percent of cancer caused by high-risk types reported for Oceania (79.0%) [48]. multiplied by lower bound estimate for percent of cancer caused by high-risk types reported for Oceania (72.3%) [48]. High: Assume 100% efficacy against high-risk subtypes based on [49], multiplied by upper bound estimate 95% confidence interval for percent of cancer caused by high-risk types reported for Oceania (79.0%) [48].

^b^Efficacy against non-severe Sp pneumonia as a proportion of efficacy against severe disease = 0.07 [50].

^c^Efficacy against non-severe RVGE as a proportion of efficacy against severe RVGE = 0.85 [51].

^d^Waning is based on values for a gamma function fitted to the efficacy by duration of follow-up from a meta-regression of randomised-controlled trials [52]. Base case uses median values, while low and high estimates are from uncertainty ranges from [52].

^e^Base case efficacy estimates for Samoa, Tonga and Tuvalu used as high estimate for Vanuatu dose 2/3 efficacy.

Abbreviations: HPVV, human papillomavirus vaccine; PCV, pneumococcal conjugate vaccine; RVGE, rotavirus gastroenteritis; RVV, rotavirus vaccine.

The UNIVAC model can capture vaccine timeliness through age-specific coverage rates. Following discussions with in-country vaccine delivery personnel, routine childhood vaccines were assumed to be delivered on time for Samoa and Tuvalu, and approximately 90% of final dose-specific coverage rates achieved at the target age and final coverage achieved by two years of age for Tonga and Vanuatu. As a result, all RVV doses were assumed to be administered within the manufacturers’ recommended age window (i.e., the first dose within 15 weeks and the last dose within 32 weeks).

Vaccine efficacy estimates were from a detailed review of the existing literature—drawing on the most recent systematic reviews and randomised controlled trials as a priority—along with feedback from experts in the field. Estimates used for vaccine efficacy (from two weeks after vaccination) and duration of protection were the same across all countries, except for RVV as rotavirus vaccines perform better in settings with low child mortality [54]. Single-dose efficacy of HPVV was based on a large-scale, randomised controlled trial in Kenya (97.5%) [47] and the estimated percent of cancer caused by high-risk subtypes in Oceania (75.7%) [48]. We assumed a lifetime duration of protection based on two previous systematic reviews of HPVV cost-effectiveness analyses [43,44]. We assume this same efficacy for one dose of the low-cost HPVV based on a recent interim analysis of a phase 3 clinical study [55]. PCV efficacy against severe pneumonia with *Streptococcus pneumoniae* (Sp) as a causative pathogen was estimated at 58% for three doses based on a Cochrane systematic review [45]. A conservative assumption of 4% PCV efficacy was estimated against non-severe Sp pneumonia following three doses [50]. We assume a five-year duration of protection for PCV (the highest risk period for children) with waning starting in the last year [46]. Initial vaccine efficacy for a full course of RVV against severe disease was estimated based on a recent meta-analysis of randomised controlled trials by child mortality strata [52]. The assumed waning of RVV efficacy was based on the same analysis [52]. Efficacy against non-severe rotavirus gastroenteritis was assumed to be 85% of the vaccine efficacy against severe disease [51]. For PCV and RVV, efficacy and waning were assumed to be the same between the lower-price and higher-price products evaluated.

### Vaccine program costs

Vaccination program costs include the costs of the vaccine dose (including freight, handling, wastage and vaccine supplies) and the incremental costs to the health system of delivering an additional vaccine in the existing immunisation program. All vaccine dose and program costs are summarised in Table 4 and Table A in S2 Appendix. Vaccine dose costs were informed by UNICEF based on PAHO Revolving Fund pricing (2017 price list). These prices reflect the assumption that all four countries introduce all three vaccines to benefit from negotiated reduced prices. In a separate analysis, we evaluated lower price vaccines, with per dose costs based on UNICEF 2022 pricing and WHO Market Information for Access to Vaccines purchase database.

**Table 4:** Program costs and healthcare costs (all in 2019 USD). See Appendix for supplies, wastage, and other costs applied to the vaccine dose costs.

	Base case values (low estimate, high estimate)				Source/Notes
	Samoa	Tonga	Tuvalu	Vanuatu	
**Vaccine program costs**					
Vaccine dose cost (excl. supplies, wastage and other charges)					
HPVV (all countries)^a^	12.00 (11.00, 14.00)				UNICEF-informed estimate
PCV (all countries)^a^	16.00 (14.50, 18.00)				UNICEF-informed estimate
RVV (all countries)^a^	7.50 (6.50, 9.00)				UNICEF-informed estimate
Lower price HPVV (all countries)	9.23 (2.90, 47.70)				See note^b^
Lower price PCV (all countries)	4.00 (3.05, 7.00)				Base/high: UNICEF pricing catalogue; Low: Gavi pricing;
Lower price RVV (all countries)	1.15 (0.85, 2.00)				Base/high: UNICEF pricing catalogue; Low: Gavi pricing;
Incremental health system costs per dose (base case, low estimate, high estimate years 1–5/high estimate years 6+)					
HPVV	2.73 (0.27, 17/5.00)	11.42 (3.76, 23/6.00)	22.65 (6.19, 134/49)	8.52 (3.34, 19/5.00)	Base and low: See note^c^; High: country specific budget^d^
PCV and RV	1.93 (0.19, 7.00/2.00)	8.08 (2.66, 9.00/2.00)	16.02 (4.38, 54/20)	6.03 (2.36, 8.00/2.00)	Base and low: See note^e^; High: country specific budget^d^
**Healthcare costs**					
Cost per outpatient visit					
AOM/non-severe pneumonia	8.52 (6.53, 11.47)	7.89 (6.06, 10.60)	8.12 (6.15, 10.87)	7.12 (5.49, 9.55)	Authors’ calculations. S2 Appendix
Severe pneumonia/meningitis, NPNM, meningitis sequelae	17.04 (13.07, 22.93)	15.78 (12.12, 21.20)	16.24 (12.29, 21.75)	14.24 (10.97, 19.10)	Assume 2 × non-severe
RVGE	7.94 (5.96, 11.65)	7.31 (5.49, 10.03)	7.68 (5.76, 11.29)	6.55 (4.91, 9.73)	Authors’ calculations. S2 Appendix
Cost per hospitalisation					
Local cancer	16,557 (8,278; 24,835)	7,059 (3,068, 7,649)	26,281 (13,141, 38,422)	578 (289, 867)	Authors’ calculations. See S2 Appendix
Regional cancer	1,845 (923, 2,768)	961 (680, 2,675)	26,281 (13,141, 39,422)	1,219 (609, 1,828)	Authors’ calculations. See S2 Appendix
Distant cancer	977 (423, 2,001)	913 (680, 1,872)	13,373 (3,029; 19,959)	835 (363, 1,712)	Authors’ calculations. See S2 Appendix
Severe pneumonia	211 (172, 749)	183 (137, 616)	262 (173, 808)	153 (124, 543)	Authors’ calculations. See S2 Appendix
Meningitis, NPNM	687 (558, 2,442)	539 (424, 1,879)	532 (393, 1,767)	398 (323, 1,413)	Authors’ calculations. See S2 Appendix
RVGE	216 (176, 763)	185 (150, 655)	167 (113, 516)	177 (144, 625)	Authors’ calculations. See S2 Appendix
Intussusception	659 (586, 2,346)	603 (534, 2,149)	312 (470, 1,881)	535 (476, 1,906)	Authors’ calculations. See S2 Appendix

^a^Price reflects assumptions that all four countries introduce all three vaccines. If one or more country decides to only introduce one or two of the vaccines, this price is not guaranteed and will likely be increased.

^b^Base case estimate from World Health Organization Market Information for Access vaccine purchase database[56]. Low estimate: UNICEF pricing catalogue. High estimate: based on pricing used in other cost-effectiveness analyses.

^c^Base case: country-specific point estimates from Portnoy and colleagues [57] multiplied by 1.4 (ratio of average costs of HPV delivery to average costs of DTP delivery in middle income countries from Immunization Delivery Cost Catalogue [58]; Low: lower 95% uncertainty estimate from Portnoy and colleagues multiplied by 1.4.

^d^High: Estimates from country-specific ADB budgets for vaccine roll out (see S1 Table for more detail). Using UNICEF-informed assumptions, a proportion from 30% to 100% of each cost component was assigned to the 3 new vaccines introduction, and costs were annualised over the component’s lifetime (assuming 5 or 10 years based on UNICEF-informed assumptions), and divided by the number of vaccine doses administered, in order to obtain an annual incremental program cost per dose. We assumed 50% of incremental costs of new vaccine introduction are allocated to HPVV doses, and 50% allocated to RVV/PCV doses together. Recurrent delivery cost per dose was added to this, from the cost of delivery per child based on Vanuatu Consolidated Budget Plan for EPI at Province Level, using costs compiled from health facility microplanning budget collected in 2014. Recurrent delivery/outreach costs calculated to be $0.46 per dose for Vanuatu. Based on UNICEF-informed estimates, recurrent delivery/outreach costs for Tonga/Samoa estimated at half that of Vanuatu ($0.23) and Tuvalu at midway between Tonga/Samoa and Vanuatu ($0.35).

^e^Base case: country-specific point estimates from Portnoy and colleagues [57]. Low: lower 95% uncertainty estimate from Portnoy and colleagues.

Abbreviations: HPVV, human papillomavirus vaccine; NPNM, non-pneumonia non-meningitis invasive diseases; PCV, pneumococcal conjugate vaccine; RVGE, rotavirus gastroenteritis; RVV, rotavirus vaccine.

The costs of other vaccine supplies (including syringes and safety boxes/bags), international handling and delivery charges, and wastage were based on UNICEF-informed estimates as outlined in Table A of S2 Appendix. We assumed a 10% wastage rate for single dose vial vaccines, whereas lower price RVV were assumed to be a 5-dose vial presentation with 80% wastage in Tuvalu due to its small population and 50% wastage in other countries based on consultation with vaccine program managers with experience in these settings (wastage was applied only to vaccine doses and not other consumables).

Incremental health systems costs per dose were included in the model to represent the additional start-up cost to the health system of introducing new vaccines plus recurrent delivery costs. This included cold chain strengthening, supply chain management training specialist, cold chain training specialist, EPI training including material development, printing of child health card, EPI/cold chain policy development and printing, Effective Vaccine Management Assessment, communications, project support, and monitoring and evaluation (Table 4 and S1 Table). For RVV and PCV, we used country-specific estimates from the published literature [57]. These estimates were largely higher than those published in the Immunization Delivery Cost Catalogue [59], however were considered to more accurately reflect local contextual factors (such as GDP, population, and the existing routine vaccination schedule). For HPVV delivery costs, we inflated the RVV and PCV delivery costs by a factor of 1.4 based on the difference in delivery costs between DTP3 and HPVV for middle income countries reported in the Immunization Delivery Cost Catalogue. We did not include any savings in incremental health system costs that might be possible from introducing multiple vaccines at once.

### Healthcare costs

For PCV and RVV, healthcare utilisation and costs are calculated from the onset of disease through to the age of five, except for pneumococcal meningitis sequelae, where healthcare costs are calculated over a lifetime horizon. For HPVV, healthcare utilisation and costs are calculated from the onset of disease throughout a lifetime horizon. For simplicity, all costs are assumed to occur in the first year of cancer diagnosis. The numbers of outpatient visits and hospitalisations are estimated multiplying number of cases by an assumed average number of visits and admissions per case of each disease. Healthcare costs per outpatient visit and hospitalisation were from mixed sources, including national or regional-level data and costing studies, and the published literature. We used WHO-CHOICE (WHO Choosing Interventions that are Cost Effective) 2011 country-specific estimates for unit costs of hospital and outpatient visits and hospital bed days. Laboratory and pharmacy costs were from in-country fee schedules where available. Unit costs per visit and hospitalisation were moderated down by assumed rates of care-seeking (see Table 1 for care-seeking assumptions). Full details are available in Table 4 and S2 Appendix.

A societal perspective was explored by including both direct costs (transportation and user-fees where applicable) and indirect costs (productivity losses for caregivers and women undergoing treatment for cervical cancer). Productivity losses were estimated by multiplying average length of hospital stay by country-specific female labour force participation rate from World Bank Data [60]. Lost days of work were valued based on country-specific GDP per capita. Productivity losses due to death were not included in the model, and as a result our findings will represent a conservative estimation of the societal costs.

### Cost-effectiveness

An incremental cost-effectiveness ratio (ICER), presented in terms of USD per DALY averted, was calculated for each vaccine compared to the status-quo (no vaccine), and a joint ICER was calculated for introduction of all three vaccines together compared to the no vaccine scenario.

Because explicit country-specific or regional cost-effectiveness thresholds are not available in the Pacific, we compared ICERs with two potential threshold estimates. As an upper estimate, we used willingness to pay thresholds of 1× GDP per capita (2019: Samoa $4,030; Tonga $3,749; Tuvalu $3,084; Vanuatu $2,861), based on historical recommendations of WHO Commission on Macroeconomics and Health [61,62]. This was the threshold primarily considered by decision-makers at the time when this analysis was used to support policy. However, WHO’s Commission on Macroeconomics and Health threshold does not account for the opportunity costs of health spending (i.e., the value of forgone benefits) in highly budget-constrained settings. As a lower estimate, we used recent estimates of cost-effectiveness thresholds of 0.61×, 0.44× and 0.27× GDP per capita in Samoa, Tonga and Vanuatu, respectively [63]. As estimates for Tuvalu were not available in this study, we used willingness to pay thresholds from Samoa as a proxy. These thresholds are within the ranges of indicative opportunity cost-based thresholds previously reported for all four countries [64].

### Uncertainty analysis

We conducted one-way and probabilistic sensitivity analyses to account for the considerable uncertainty surrounding underlying epidemiological and cost data and test the robustness of our results to changes in key input parameters and model choices. Upper- and lower-bound estimates were explored across estimates of disease event rates—which included incidence and mortality—(Table 1), disability weights and durations (Table D in S1 Appendix; explored in the probabilistic sensitivity analysis only), vaccine coverage rates (Table 2), vaccine efficacy and duration (Table 3), and program and healthcare costs (Table 4 and S2 Appendix).

For the one-way sensitivity analysis of discount rate, we ran a scenario with costs discounted at 3% and ICERs calculated using undiscounted DALYs averted in line with recent WHO guidance on discount rates [65]. We ran another scenario where both costs and DALYs were discounted at 6% (ADB-recommended discount rate for social sector projects).

For the probabilistic sensitivity analysis, there were limited data to inform the true underlying distributions for most parameters and the extent to which each of the parameters are correlated. We therefore assumed simple PERT-Beta distributions for each parameter as others have done with UNIVAC [19,66], and ran the model 1,000 times for individual vaccines in each country. Although this is a simplification, the PERT-Beta distribution is able to reproduce plausible distributions that reflect the mid, low, and high values specified for each parameter, including right-skewed and left-skewed distributions. Dose prices were fixed in probabilistic analyses [67]. For each draw, costs were summed across the three vaccines and likewise for DALYs averted to give results for the full program. Results from each draw were directly plotted on cost-effectiveness planes to generate cost-effectiveness clouds rather than point estimates. To generate cost-effectiveness acceptability curves, we calculated the proportion of draws with ICERs below increasing willingness to pay thresholds, with thresholds increasing in 10 USD increments until probability approached 1.

### Budget impact analysis

We estimated the budget impact of the new vaccine introductions from a government perspective, factoring in development partner support. Government share of dose costs began at 0% in year one and increased by 20 percentage points each year over the first five years until the full vaccine dose cost was paid by the government. Over the first five years, we modelled 50% of the incremental health system costs of vaccine introduction to be paid by government, increasing to 100% for years 6 to 10. We present annual undiscounted vaccine program costs for the base case and lower price vaccines separately by country, vaccine, and payer (donor or government), alongside averted healthcare costs over a 10-year period. Both financial (program costs) and economic (healthcare costs averted) costs were included in the budget impact analysis. We also present results in terms of the increase in total health expenditure and immunisation budget (undiscounted) based on year six when the full cost of the program is paid by the government.

## Results

### Base case scenario

**Fig 2:**
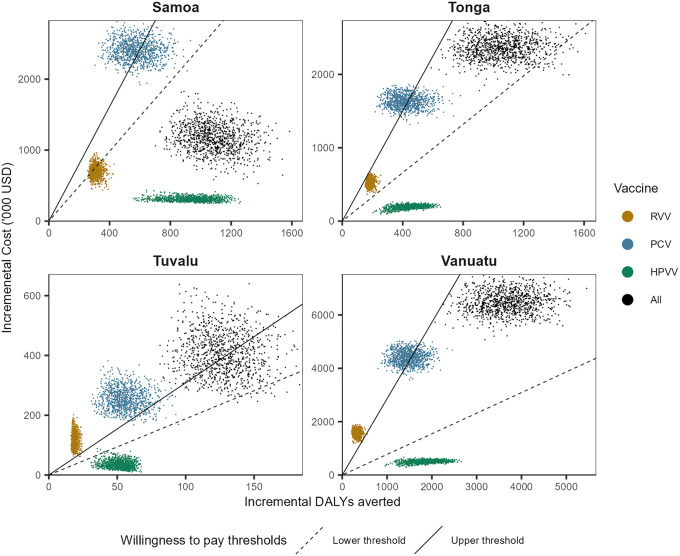
Costs and DALY averted results from probabilistic sensitivity analysis of each vaccine and the combined program in each country. Solid black line: Upper WTP estimate is 1× GDP per capita in each country. Dashed black line: Lower WTP estimate is 0.61×, 0.44×, 0.61× and 0.27× GDP per capital in Samoa, Tonga, Tuvalu and Vanuatu, respectively. Abbreviations: DALY, disability-adjusted life year; HPVV, human papillomavirus vaccine; PCV, pneumococcal conjugate vaccine; RVV, rotavirus vaccine; USD, United States dollars; WTP, willingness to pay.

Vaccine program costs, healthcare cost savings to government, and health benefits of a 10-year vaccine program adopting HPVV, PCV and RVV at PAHO Revolving Fund pricing are shown in Table 5. The costs per DALY averted (compared to the status quo) for these vaccines are shown in Fig 2. See Table 1 and Fig 1 in S3 Appendix for the same data for the lower price vaccines.

**Table 5:** Base case cost-effectiveness results for HPVV, PCV and RVV. ICERs for each vaccine and all three vaccines are expressed as cost per DALY averted compared to the status quo (10-year program, lifetime benefit stream). In the primary analysis, future costs and benefits were discounted at 3%. Results are also shown for a separate analysis where only costs were discounted (3%) and future benefits were not discounted in line with WHO guidelines [65].

	Samoa	Tonga	Tuvalu	Vanuatu
HPVV				
Vaccine program costs (USD)	316,091	249,570	36,385	618,217
Healthcare cost savings (USD)	20,615	6,171	9,849	31,803
Hospitalisations averted	43	22	2	129
Deaths averted	219	124	13	530
DALYs averted	1,036	556	58	2,267
ICER (USD/DALY averted)	285	437	456	257
PCV				
Vaccine program costs (USD)	2,611,339	2,020,523	259,463	5,239,775
Healthcare cost savings (USD)	124,893	66.627	7,764	204,474
Outpatient visits averted	1,878	1,987	106	3,672
Hospitalisations averted	488	312	28	1,204
Deaths averted	23	16	2	60
DALYs averted	622	421	56	1,582
ICER (USD/DALY averted)	3,997	4,646	4,479	3,184
RVV				
Vaccine program costs (USD)	922,469	822,047	121,689	2,061,196
Healthcare cost savings (USD)	167,579	100,768	7,667	128,773
Outpatient visits averted	10,736	5,227	501	9,560
Hospitalisations averted	502	424	30	482
Deaths averted	11	6	1	10
DALYs averted	297	176	18	265
ICER (USD/DALY averted)	2,542	4,102	6,300	7,284
All three vaccines				
ICER (USD/DALY averted)	1,809	2,531	2,972	1,836
ICER as a percentage of GDP (2019)	42%	58%	73%	60%
Societal ICER (USD/DALY averted)	1.779	2,500	2,950	1,801
Societal ICER as a percentage of GDP (2019)	41%	57%	73%	59%
ICER at lower discount rate (USD/DALY averted)	403	605	734	416
ICER at lower discount rate as a percentage of GDP (2019)	9%	14%	18%	14%

Abbreviations: DALY, disability-adjusted life year; GDP, gross domestic product; HPVV, human papillomavirus vaccine; ICER, incremental cost-effectiveness ratio; PCV, pneumococcal conjugate vaccine; RVV, rotavirus vaccine; USD, United States dollars.

Simultaneous introduction of all three vaccines in all four countries could prevent 1,015 premature deaths over the lifetimes of the people vaccinated in the 10-year program (886 cervical cancer deaths, 101 pneumococcal deaths, 28 rotavirus deaths), equivalent to just over half of the deaths that would otherwise have been caused by these diseases. Over 33,000 outpatient visits and 3,600 hospitalisations would be averted across the four countries. Due to the assumption of timely administration of vaccines in each country, excess cases of intussusception would be negligible (<1 over the 10-year vaccination program).

In the base case analysis, the ICER for HPVV falls just at or below 0.10× GDP per capita in all countries. The ICERs for PCV and RVV mostly fall between 1× and 1.5× GDP per capita in all countries except for RVV in Samoa (ICER of 0.59× GDP per capita) and RVV in Vanuatu (ICER of 2.3× GDP per capita).

Considering all three vaccines introduced together at PAHO Revolving Fund prices, the joint ICER (compared to the current status without these vaccines) ranged from 0.43× GDP per capita in Samoa to 0.73× GDP per capita in Tuvalu. The secondary analysis conducted from a societal perspective resulted in slightly more favourable results (lower ICERs) but minimal difference in findings.

ICERs of the lower price vaccines were mostly more favourable than for the PAHO Revolving Fund-priced vaccines, ranging from 0.05× GDP per capita (for HPVV in Samoa) to 1.9× GDP per capita (for RVV in Tuvalu). The ICER for the lower-priced RVV was higher than for the higher-price product in Tuvalu owing to the three-dose schedule and the high per-dose delivery costs (due to the high wastage rate). The simultaneous introduction of all three lower-priced vaccines had an ICER ranging from 0.15× GDP per capita in Samoa to 0.58× GDP per capita in Tuvalu.

### Sensitivity analysis

**Fig 3:**
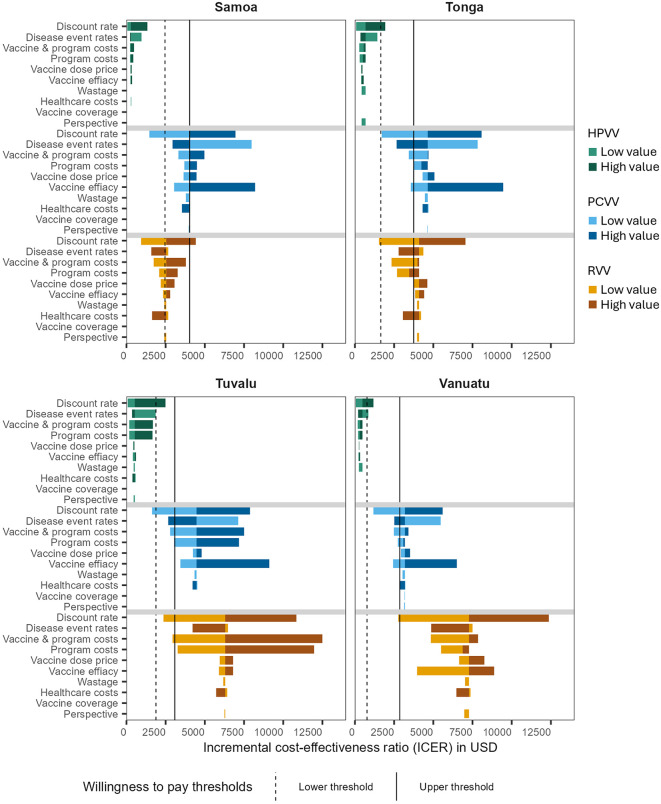
One-way sensitivity analysis of the incremental cost-effectiveness ratios for each vaccine in each country. Disease event rates include upper and lower estimates for both incidence and mortality. Vaccine efficacy includes upper and lower estimates for both efficacy and duration of effect. Upper and lower estimates of vaccine dose price represent the range of quoted prices from different suppliers of the same vaccine. “Upper” estimate for the perspective parameter represents the societal perspective. Solid black line: Upper WTP estimate is 1× GDP per capita in each country. Dashed black line: Lower WTP estimate is 0.61×, 0.44×, 0.61× and 0.27× GDP per capital in Samoa, Tonga, Tuvalu and Vanuatu, respectively. Abbreviations: DALY, disability-adjusted life year; HPVV, human papillomavirus vaccine; PCV, pneumococcal conjugate vaccine; RVV, rotavirus vaccine; USD, United States dollars; WTP, willingness to pay.

Tornado diagrams for the one-way sensitivity analysis are shown in Fig 3, with diagrams for the lower price vaccines shown in Fig B of S3 Appendix. Due to the preventive nature of these vaccine programs, with health benefits accruing well into the future, the choice of discount rate had the greatest impact on results. When discounting (3%) was only applied to costs, but not benefits, the ICER for the combined program ranged from 0.09 to 0.14× GDP per capita per DALY averted in Samoa and Tuvalu, respectively (Table 5).

Estimates of incidence of disease, mortality rates, and vaccine efficacy were highly influential inputs affecting study findings. Vaccine program costs were highly influential parameters, particularly in Tuvalu, where the cost per DALY averted approached 2× GDP per capita for PCV and exceeded 3× GDP per capita for RVV at the higher parameter estimates. This finding is largely driven by the high estimated costs of immunisation programs in Tuvalu, where the population is smaller and more geographically dispersed on outer islands compared to the other three countries. ICERs for HPVV were favourable across most parameter estimates evaluated, falling just over, or well below the lower estimate of willingness to pay thresholds in each country. Payer perspective (government or societal) and vaccine coverage estimates had the least effect on cost-effectiveness for all vaccines in all countries.

**Fig 4:**
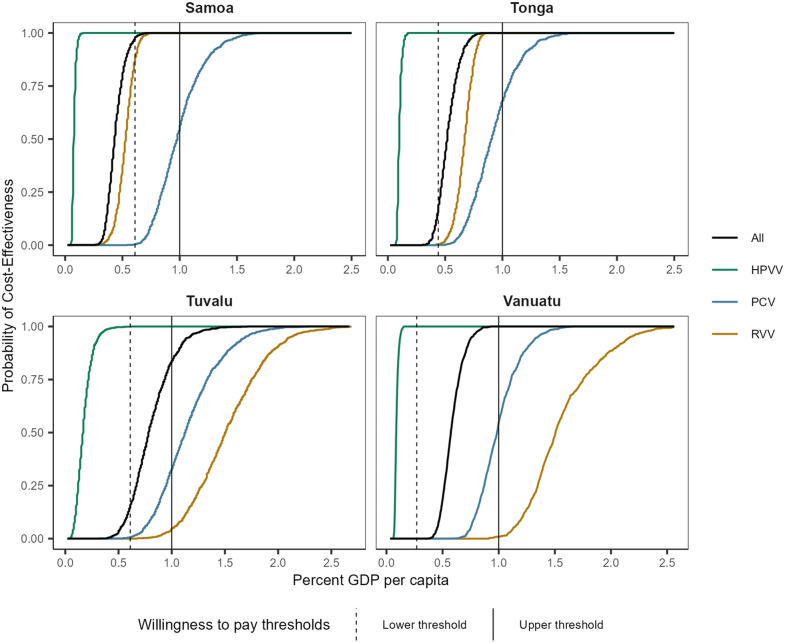
Cost-effectiveness acceptability curves for each vaccine and the combined program in each country. Upper WTP estimate is 1× GDP per capita in each country. Lower WTP estimate is 0.61×, 0.44×, 0.61× and 0.27× GDP per capital in Samoa, Tonga, Tuvalu and Vanuatu, respectively. GDP, gross domestic product; HPVV, human papillomavirus vaccine; PCV, pneumococcal conjugate vaccine; RVV, rotavirus vaccine; USD, United States dollars.

The acceptability curves from the probabilistic sensitivity analyses are shown in Fig 4, with curves for for lower price vaccines shown in Fig C of S3 Appendix. At the lower estimate of willingness to pay, HPVV is highly likely to be cost-effective in all countries (probabilities >0.99), while the probability of PCV and RVV being cost-effective at this willingness to pay threshold is much lower (<0.006) in all countries except Samoa, where the probability of RVV being cost-effective is 0.877. At this willingness to pay threshold, simultaneous introduction of all three vaccines has a probability of being cost-effective of 0.973 in Samoa, and less than 0.2 in the other three countries. If willingness to pay is 1× GDP per capita, the probability that introduction of all three vaccines is cost-effective is greater than 0.846 in all countries.

### Budget impact

**Fig 5:**
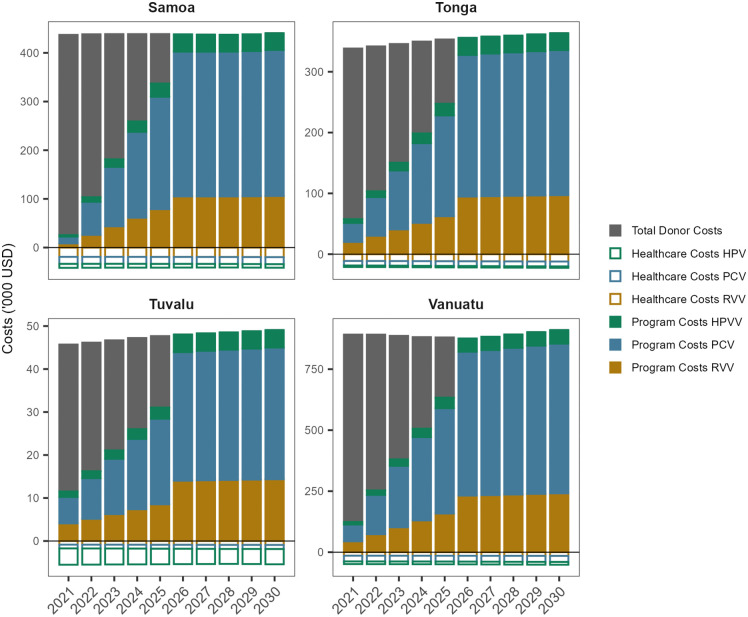
Results from budget impact analysis for each vaccine in each country. Underlying budget impact data can be found in S3 Appendix Table B. HPVV, human papillomavirus vaccine; PCV, pneumococcal conjugate vaccine; RVV, rotavirus vaccine; USD, United States dollars.

Results from the budget impact analysis are shown in Fig 5 for the vaccines at PAHO Revolving Fund pricing and Fig D of S3 Appendix for the lower price vaccines. Underlying budget impact data can be found in S3 Appendix Table B (PAHO Revolving Fund priced vaccines) and Table C (lower priced vaccines).

Addition of the three vaccines would increase the cost of the immunisation program by 438,098 USD, 355,744 USD, 48,029 USD and 875,589 USD in Samoa, Tonga, Tuvalu and Vanuatu, respectively, after scaling up the full cost of the program to the government at year six. This would represent an increase in total health expenditure ranging from 0.45% in Tuvalu to 2.76% in Vanuatu each year. The annual increase in spending on vaccines would range from 359% in Samoa to 1,368% in Vanuatu. When considering the reduction in spending on healthcare (by reducing disease burden), the increase spending on vaccines would range from approximately 326% in Samoa to 1,290% in Vanuatu. If lower price vaccines were introduced, the annual impact on government budgets (at year six) would be 182,254 USD, 217,013 USD, 39,177 USD and 496,185 USD for the governments of Samoa, Tonga, Tuvalu and Vanuatu, respectively (representing an increase in vaccine budgets ranging from 149% in Samoa to 775% in Vanuatu).

## Discussion

This is the first study to our knowledge to provide evidence on the cost-effectiveness of three new vaccines under consideration for introduction into routine immunisation schedules simultaneously in four Pacific Island countries, with development partner support. Simultaneous introduction of all three vaccines was the policy decision these countries were faced with when this work was commissioned as the high financial and human cost of negotiating budgets, prices, and vaccine introduction activities in these settings made it necessary to consider introduction of all three vaccines or no vaccine at all. The findings of our study allow for consideration of the value of concurrent introduction of multiple vaccines, enabling favourable pricing negotiations and efficiencies in vaccine introduction activities. Our analysis found that introduction of HPVV, RVV and PCV together at PAHO Revolving Fund prices has an ICER of around half to three-quarters of GDP per capita in each country. Policy-makers—who had access to non-fungible funding for vaccine introduction at the time of decision-making—considered this good value for money (based on a threshold of 1× GDP per capita per DALY averted). This evidence formed part of a package of information guiding country-level decisions to introduce all three vaccines in these four countries. While the initial costs of introduction (purchase of vaccine doses and community mobilisation costs) were offset by financing support from ADB and UNICEF, all four country governments committed to progressively transitioning to 100% financing of the vaccination program.

Recognising that a threshold of 1× GDP per capita is suggested to be too high, particularly for LMICs, we also compared ICERs to thresholds that consider the opportunity costs of investing in a health intervention (and the benefits forgone by not investing those resources elsewhere) [63]. Based on these lower thresholds (available for Samoa, Tonga and Vanuatu), introduction of these three vaccines is still likely to be cost-effective in Samoa, but may exceed willingness to pay in Tonga and Vanuatu. In Tuvalu, the program ICER exceeds the most optimistic of these recently reported thresholds for these countries (Samoan threshold of 0.61× GDP per capita). Lower-priced vaccines have a higher likelihood of being cost-effective at these more conservative estimates of willingness to pay, with costs per DALY averted ranging from 0.05× GDP per capita in Samoa to 0.58× GDP per capita in Tuvalu when all three vaccines are introduced. However, it should be noted that some lower-priced vaccines place an increased burden on immunisation programs (e.g., the lower-priced RVV requires a three-dose schedule and is presented in a multidose vial, leading to higher wastage and requiring more resources for delivery). Beyond assessing cost-effectiveness against a specific threshold, decision-makers must be guided by several considerations, including program feasibility and budget impact, when selecting public health interventions [68].

Our analysis estimated that vaccine adoption would require substantial increases in vaccine budgets ranging from 359% in Samoa to over 1,300% in Vanuatu. Even with the adoption of lower-priced products, vaccine budgets would need increases of between 149% (Samoa) to 775% (Vanuatu). The introduction of vaccines has been slow in middle-income countries which are not eligible for Gavi funding support, with vaccine price being a major barrier to introduction [69]. Through pooled procurement, the VII has facilitated access to vaccine prices that are more affordable than what countries with small populations may be able to negotiate on their own terms. This has been reflected in our analysis, and higher dose prices with less favourable cost-effectiveness results may be expected if only a subset of vaccines were adopted by a few countries. However, more needs to be done to ensure middle-income countries, including small island countries in the Pacific, have access to affordable vaccines [70].

There are limitations to our analyses that should be noted. For many disease burden inputs we drew on data from Fiji, where surveillance systems are comparatively more developed. This enabled access to a larger and more robust body of evidence than what is available for Samoa, Tonga, Tuvalu and Vanuatu. We consider this to be reasonable, as Fiji shares several health, demographic and economic features in common with the four countries of this analysis. Similarities in results between countries will, in part, be driven by reliance on common data for modelling disease burden. Likewise, differences can be attributed to a smaller number of inputs where country-specific data were used (such as case fatality ratio for severe pneumonia and diarrhoea). Disease incidence and mortality estimates were among the most influential parameters impacting ICERs. Improved epidemiological data would provide more certainty on vaccine cost-effectiveness and improve capacity for evidence-based decision-making in these countries but would require significant investment to strengthen disease surveillance systems.

We make several assumptions and model design choices that result in conservative cost-effectiveness estimates. The discount rate used in our base case analysis is conservative (3% for both costs and benefits) compared to current WHO recommendations of leaving benefits undiscounted while costs are discounted at 3% (which we used as the lower bound for our sensitivity analysis) [65]. Discount rate was one of the largest drivers of cost-effectiveness and the combined vaccination program was cost-effective in all countries at WHO-recommended discount rates, even at lower willingness to pay thresholds. Secondly, our use of WHO-CHOICE estimates for unit costs of hospitalisation and outpatient visits represents a conservative assumption, as these estimates often underestimate the true costs of these services [71]. Further, we assume no cases of rotavirus gastroenteritis in children over the age of two years, despite evidence from Fiji suggesting that 16% of hospitalised cases in under five year olds are among those over the age of two [6]. While our assumption underestimates the burden of rotavirus disease, it has minimal impact on the overall ICER (~1% change in ICER).

Modelling a three-dose schedule of PCV may overestimate the potential ICER and budget impact of this vaccine in light of emerging evidence suggesting that a two-dose PCV schedule may offer comparable protection to a three-dose schedule [72,73]. Additionally, broader value elements of vaccines, such as those articulated in the Full Value of Vaccines Assessments framework, have not been included [74].

It should be noted that the model has been updated to incorporate more robust data that have become available since the initial analysis was used to support decision-making. Updates included modelling a single dose schedule for HPVV (in line with updated WHO recommendations). Additionally, the health system costs of vaccine delivery were updated with recent country-specific estimates that are higher than those originally used but considered to more accurately reflect the high costs of programmatic activities in these settings [57]. The health system delivery cost estimates from this source are modelled based on several primary studies, some of which include start-up as well as recurrent costs. We applied the modelled estimates to each year across the 10-year program modelled, which likely overestimates program costs given that start-up costs would not continue past the first few years of introduction. Our high estimate, which was informed by review of program budgets and advice from immunisation experts, suggests that health system delivery costs may reduce to approximately 20%–35% after the first five years, once start-up costs are no longer incurred. Therefore, use of these updated delivery costs has resulted in more conservative estimates of cost-effectiveness.

Further limitations of our analysis should be noted. Firstly, the costs and health impacts of catch-up campaigns were not included in this evaluation. Vaccine-specific catch-up programs for certain age groups were under consideration for some of the countries alongside routine introduction, but were ultimately not implemented. Next, we do not consider any differential health impacts compared to a single vaccine, as these are likely to be negligible. Further, we valued lost days of work based on formal labour force participation, which does not account for unpaid time and underestimates opportunity costs attributable to those not in the formal labour force. Finally, the limitations of using a static model should be acknowledged. Dynamic transmission models allow for capture of potential indirect effects, including both herd immunity and serotype replacement, which can be particularly significant for some vaccines. The recent literature indicates little difference between static and dynamic models in some circumstances, including when evaluating the cost-effectiveness of RVV with a focus on outcomes in children under 5 years, and HPV vaccination of adolescent girls [66,75–78]. For PCV, the potential for indirect effects (herd immunity and serotype replacement) is much greater than for rotavirus and HPV vaccines, thus a dynamic model could demonstrate different results compared to a static model [79–81]. While a recently published pseudo-dynamic model of PCV impact generated similar estimates to UNIVAC when using the same coverage scenarios, the study noted reduced potential for herd effects in LMICs compared to high income countries based on post-licensure data from a very small number of countries (Kenya, The Gambia, Israel, England, Lao PDR, Mongolia), and the potential for serotype replacement, potentially making results more conservative [82]. However, the LMICs with post-licensure data may not be representative of these Pacific Island nations. With respect to serotype replacement, studies have reported no evidence of a change in non-vaccine-serotype invasive pneumococcal disease among children under five years in the 3–5 years following PCV10 introduction in Fiji [79]. We would expect this to be somewhat similar in these four Pacific Island countries. While we acknowledge that it remains unclear whether a dynamic modelling framework would lead to results which are more or less cost-effective, we believe the findings are unlikely to vary significantly. Developing a dynamic transmission model and collecting the data necessary to calibrate it was beyond the scope of the commissioned analysis and would not have been feasible within the policy window when introduction of these vaccines was being considered (which occurred just prior to and during the COVID-19 pandemic).

The findings from this study suggest that HPVV, RVV and PCV introduced together would have been cost-effective in Samoa, Tonga, Tuvalu and Vanuatu using historic cost-effectiveness thresholds that policy-makers applied at the time of decision-making. This evidence helped inform decisions to adopt all three vaccines at prices close to PAHO Revolving Fund pricing in all four countries. However, the vaccines at these prices are unlikely to be considered cost-effective using more recent health opportunity cost-based thresholds. In contrast, introduction of lower priced HPVV, RVV and PCV together would likely be considered cost-effective within these newer opportunity cost-based thresholds. Our findings highlight the importance of ensuring that lower price vaccines are accessible to middle-income countries which are ineligible for Gavi financing. In addition to affordable vaccine pricing, Ministries of Health and Ministries of Finance must work closely together to ensure sufficient fiscal space for health programs to continue to meet the population’s health needs and expectations.

## Supporting information

S1 Text Country context. (DOCX)

S2 Text UNIVAC model description. (DOCX)

S1 Checklist CHEERS Checklist (https://doi.org/10.1016/j.jval.2021.10.008). (DOCX)

S1 Appendix Input parameters for estimating disease burden. (DOCX)

S2 Appendix Input parameters for estimating costs. (DOCX)

S3 Appendix Results for cost-effectiveness analysis of lower price vaccines. (DOCX)

S1 Table Calculation of upper estimates for incremental health system costs. (XLSX)

## Abbreviations

ADB: Asian Development Bank
DALYs: disability-adjusted life years
DTP: diphtheria–tetanus–pertussis
ICER: incremental cost-effectiveness ratio
GBD: Global Burden of Disease
GDP: gross domestic product
HPVV: human papillomavirus vaccine
LCU: local currency units
LMICs: low- and middle-income countries
MCEE: Maternal and Child Epidemiology Estimation collaboration
NPNM: non-pneumonia non-meningitis invasive diseases
PAHO: Pan American Health Organization
PCV: pneumococcal conjugate vaccine
RVV: rotavirus vaccine
USD: United States dollars
VII: Vaccine Independence Initiative
WHO: World Health Organization
WUENIC: WHO/UNICEF Estimates of National Immunization Coverage

## References

[1] World Health Organization. Rotavirus vaccines; WHO position paper – July 2021. Wkly Epidemiol Rec. 2021;:301–20.

[2] Tate JE, Burton AH, Boschi-Pinto C, Parashar UD, World Health Organization–Coordinated Global Rotavirus Surveillance Network. Global, regional, and national estimates of rotavirus mortality in children <5 years of age, 2000-2013. Clin Infect Dis. 2016;62 Suppl 2(Suppl 2):S96–105. doi: 10.1093/cid/civ1013 27059362 PMC11979873

[3] World Health Organization. Pneumococcal conjugate vaccines in infants and children under 5 years of age: WHO position paper – February 2019. Weekly Epidemiol Record. 2019;94(8):85–104.

[4] World Health Organization. Cervical cancer elimination initiative. Global strategy to accelerate the elimination of cervical cancer as a public health problem. World Health Organization; 2020. p. 52.

[5] Vince JD. Millennium Development Goals: progress in Oceania. Arch Dis Child. 2015;100 Suppl 1:S63–5. doi: 10.1136/archdischild-2013-305618 25613974

[6] Jenney AWJ, Reyburn R, Ratu FT, Tuivaga E, Nguyen C, Covea S, et al. The impact of the rotavirus vaccine on diarrhoea, five years following national introduction in Fiji. Lancet Reg Health West Pac. 2020;6:100053. doi: 10.1016/j.lanwpc.2020.100053 34327400 PMC8315333

[7] Lai J, Nguyen C, Tabwaia B, Nikuata A, Baueri N, Timeon E, et al. Temporal decline in diarrhea episodes and mortality in Kiribati children two years following rotavirus vaccine introduction, despite high malnutrition rates: a retrospective review. BMC Infect Dis. 2020;20(1):207. doi: 10.1186/s12879-020-4874-6 32164562 PMC7069014

[8] Reyburn R, Tuivaga E, Nguyen CD, Ratu FT, Nand D, Kado J, et al. Effect of ten-valent pneumococcal conjugate vaccine introduction on pneumonia hospital admissions in Fiji: a time-series analysis. Lancet Glob Health. 2021;9(1):e91–8. doi: 10.1016/S2214-109X(20)30421-6 33227258

[9] Reyburn R, Tuivaga E, Ratu T, Young S, Garland SM, Murray G, et al. A single dose of quadrivalent HPV vaccine is highly effective against HPV genotypes 16 and 18 detection in young pregnant women eight years following vaccination: an retrospective cohort study in Fiji. Lancet Reg Health West Pac. 2023;37:100798. doi: 10.1016/j.lanwpc.2023.100798 37359996 PMC10285272

[10] The World Bank and GAVI Alliance. Brief 13: UNICEF—vaccine Independence Initiative (VII). Immunization Financing Toolkit: The World Bank and GAVI Alliance; 2010.

[11] Asian Development Bank. Systems Strengthening for Effective Coverage of New vaccines in the Pacific project under the Asia Pacific Vaccine Access Facility (Additional Financing) [cited 2024]. Available from: https://www.adb.org/projects/50282-003/main

[12] Bureau régional pour les Amériques de l’Organisation mondiale de la Santé. PAHO Revolving Fund [Oct 2024]. Available from: https://www.paho.org/fr/node/89075

[13] Reyburn R, Tsatsaronis A, von Mollendorf C, Mulholland K, Russell FM, ARI Review Group. Systematic review on the impact of the pneumococcal conjugate vaccine ten valent (PCV10) or thirteen valent (PCV13) on all-cause, radiologically confirmed and severe pneumonia hospitalisation rates and pneumonia mortality in children 0-9 years old. J Glob Health. 2023;13:05002. doi: 10.7189/jogh.13.05002 36734192 PMC9896304

[14] Jauregui B, Janusz CB, Clark AD, Sinha A, Garcia AGF, Resch S, et al. ProVac Global Initiative: a vision shaped by ten years of supporting evidence-based policy decisions. Vaccine. 2015;33 Suppl 1:A21-7. doi: 10.1016/j.vaccine.2014.12.080 25919164

[15] Debellut F. Impact and cost-effectiveness of rotavirus vaccination in 73 Gavi countries. Thirteenth International Rotavirus Symposium; 29–31 August 2018; Minsk, Belarus; 2018.

[16] Anwari P, Debellut F, Pecenka C, Parwiz SM, Clark A, Groman D, et al. Potential impact and cost-effectiveness of rotavirus vaccination in Afghanistan. Vaccine. 2018;36(51):7769–74. doi: 10.1016/j.vaccine.2017.10.058 29107346 PMC6290387

[17] García Fariñas A, Linares-Pérez N, Clark A, Toledo-Romaní ME, Omeiri NE, Marrero Araújo MC, et al. Cost-effectiveness of introducing a domestic pneumococcal conjugate vaccine (PCV7-TT) into the Cuban national immunization programme. Int J Infect Dis. 2020;97:182–9. doi: 10.1016/j.ijid.2020.05.078 32474199

[18] Thobari JA, Watts E, Carvalho N, Haposan JH, Clark A, Debellut F, et al. Cost effectiveness analysis of rotavirus vaccination in Indonesia. Vaccine. 2025;43(Pt 2):126478. doi: 10.1016/j.vaccine.2024.126478 39500219

[19] Mahmud S, Baral R, Sanderson C, Pecenka C, Jit M, Li Y, et al. Cost-effectiveness of pharmaceutical strategies to prevent respiratory syncytial virus disease in young children: a decision-support model for use in low-income and middle-income countries. BMC Med. 2023;21(1):138. doi: 10.1186/s12916-023-02827-5 37038127 PMC10088159

[20] Turner HC, Lauer JA, Tran BX, Teerawattananon Y, Jit M. Adjusting for inflation and currency changes within health economic studies. Value Health. 2019;22(9):1026–32. doi: 10.1016/j.jval.2019.03.021 31511179

[21] Wahl B, O’Brien KL, Greenbaum A, Majumder A, Liu L, Chu Y, et al. Burden of *Streptococcus pneumoniae* and *Haemophilus influenzae* type b disease in children in the era of conjugate vaccines: global, regional, and national estimates for 2000-15. Lancet Glob Health. 2018;6(7):e744–57. doi: 10.1016/S2214-109X(18)30247-X 29903376 PMC6005122

[22] Lutui F, Grant CC, Best E, Howie S, Aho G. Invasive pneumococcal disease in children in Tonga. Pediatr Infect Dis J. 2017;36(2):239–40. doi: 10.1097/INF.0000000000001400 27832020

[23] Russell FM, Carapetis JR, Tikoduadua L, Paeds D, Chandra R, Seduadua A, et al. Invasive pneumococcal disease in Fiji: clinical syndromes, epidemiology, and the potential impact of pneumococcal conjugate vaccine. Pediatr Infect Dis J. 2010;29(9):870–2. doi: 10.1097/INF.0b013e3181ec7ae2 20622710

[24] O’Brien KL, Wolfson LJ, Watt JP, Henkle E, Deloria-Knoll M, McCall N, et al. Burden of disease caused by *Streptococcus pneumoniae* in children younger than 5 years: global estimates. Lancet. 2009;374(9693):893–902. doi: 10.1016/S0140-6736(09)61204-6 19748398

[25] Clark AD, Hasso-Agopsowicz M, Kraus MW, Stockdale LK, Sanderson CFB, Parashar UD, et al. Update on the global epidemiology of intussusception: a systematic review of incidence rates, age distributions and case-fatality ratios among children aged <5 years, before the introduction of rotavirus vaccination. Int J Epidemiol. 2019;48(4):1316–26. doi: 10.1093/ije/dyz028 30879038 PMC6693807

[26] Paterson JE, Carter S, Wallace J, Ahmad Z, Garrett N, Silva PA. Pacific Islands Families Study: risk factors associated with otitis media with effusion among Pacific 2-year-old children. Int J Pediatr Otorhinolaryngol. 2007;71(7):1047–54. doi: 10.1016/j.ijporl.2007.03.013 17467064

[27] Monasta L, Ronfani L, Marchetti F, Montico M, Vecchi Brumatti L, Bavcar A, et al. Burden of disease caused by otitis media: systematic review and global estimates. PLoS One. 2012;7(4):e36226. doi: 10.1371/journal.pone.0036226 22558393 PMC3340347

[28] Bilcke J, Van Damme P, Van Ranst M, Hens N, Aerts M, Beutels P. Estimating the incidence of symptomatic rotavirus infections: a systematic review and meta-analysis. PLoS One. 2009;4(6):e6060. doi: 10.1371/journal.pone.0006060 19557133 PMC2699052

[29] Jenney A, Tikoduadua L, Buadromo E, Barnes G, Kirkwood CD, Boniface K, et al. The burden of hospitalised rotavirus infections in Fiji. Vaccine. 2009;27:F108–11. doi: 10.1016/j.vaccine.2009.08.071 19931707

[30] Law I, Fong JJ, Buadromo EM, Samuela J, Patel MS, Garland SM, et al. The high burden of cervical cancer in Fiji, 2004-07. Sex Health. 2013;10(2):171–8. doi: 10.1071/SH12135 23557630

[31] Arbyn M, Weiderpass E, Bruni L, de Sanjosé S, Saraiya M, Ferlay J, et al. Estimates of incidence and mortality of cervical cancer in 2018: a worldwide analysis. Lancet Glob Health. 2020;8(2):e191–203. doi: 10.1016/S2214-109X(19)30482-6 31812369 PMC7025157

[32] GBD Results 2021 [Internet]. IHME, University of Washington; 2024 [cited Sep 2024]. Available from: https://vizhub.healthdata.org/gbd-results/

[33] Campos NG, Sharma M, Clark A, Lee K, Geng F, Regan C, et al. The health and economic impact of scaling cervical cancer prevention in 50 low- and lower-middle-income countries. Int J Gynaecol Obstet. 2017;138 Suppl 1:47–56. doi: 10.1002/ijgo.12184 28691334

[34] Mahadevan M, Navarro-Locsin G, Tan HKK, Yamanaka N, Sonsuwan N, Wang P-C, et al. A review of the burden of disease due to otitis media in the Asia-Pacific. Int J Pediatr Otorhinolaryngol. 2012;76(5):623–35. doi: 10.1016/j.ijporl.2012.02.031 22404948

[35] Russell F, Sanderson C, Temple B, Mulholland EK. Global review of the distribution of pneumococcal disease by age and region; 2011. Available from: https://www.who.int/immunization/sage/6_Russel_review_age_specific_epidemiology_PCV_schedules_session_nov11.pdf

[36] Soares-Weiser K, Maclehose H, Bergman H, Ben-Aharon I, Nagpal S, Goldberg E, et al. Vaccines for preventing rotavirus diarrhoea: vaccines in use. Cochrane Database Syst Rev. 2012;(2):CD008521. doi: 10.1002/14651858.CD008521.pub2 22336845

[37] Hasso-Agopsowicz M, Ladva CN, Lopman B, Sanderson C, Cohen AL, Tate JE, et al. Global review of the age distribution of rotavirus disease in children aged <5 years before the introduction of rotavirus vaccination. Clin Infect Dis. 2019;69(6):1071–8. doi: 10.1093/cid/ciz060 30689799 PMC6736387

[38] Ratu FT, Reyburn R, Tuivaga E, Tuiketei A, Jenkins K, Mulholland K, et al. Epidemiology of intussusception before and after rotavirus vaccine introduction in Fiji. Sci Rep. 2018;8(1):11194. doi: 10.1038/s41598-018-29515-2 30046133 PMC6060119

[39] Clark A, Tate J, Parashar U, Jit M, Hasso-Agopsowicz M, Henschke N, et al. Mortality reduction benefits and intussusception risks of rotavirus vaccination in 135 low-income and middle-income countries: a modelling analysis of current and alternative schedules. Lancet Glob Health. 2019;7(11):e1541–52. doi: 10.1016/S2214-109X(19)30412-7 31607466 PMC7024991

[40] Salomon JA, Haagsma JA, Davis A, de Noordhout CM, Polinder S, Havelaar AH, et al. Disability weights for the Global Burden of Disease 2013 study. Lancet Glob Health. 2015;3(11):e712-23. doi: 10.1016/S2214-109X(15)00069-8 26475018

[41] GBD 2017 Disease and Injury Incidence and Prevalence Collaborators. Global, regional, and national incidence, prevalence, and years lived with disability for 354 diseases and injuries for 195 countries and territories, 1990-2017: a systematic analysis for the Global Burden of Disease Study 2017. Lancet. 2018;392(10159):1789–858. doi: 10.1016/S0140-6736(18)32279-7 30496104 PMC6227754

[42] CDC. CfDCaP. The pink book. Rotavirus; 2015. Available from: https://www.cdc.gov/vaccines/pubs/pinkbook/rota.html

[43] Pink J, Parker B, Petrou S. Cost effectiveness of HPV vaccination: a systematic review of modelling approaches. Pharmacoeconomics. 2016;34(9):847–61. doi: 10.1007/s40273-016-0407-y 27178048

[44] Fesenfeld M, Hutubessy R, Jit M. Cost-effectiveness of human papillomavirus vaccination in low and middle income countries: a systematic review. Vaccine. 2013;31(37):3786–804. doi: 10.1016/j.vaccine.2013.06.060 23830973

[45] Lucero MG, Dulalia VE, Nillos LT, Williams G, Parreño RAN, Nohynek H, et al. Pneumococcal conjugate vaccines for preventing vaccine-type invasive pneumococcal disease and X-ray defined pneumonia in children less than two years of age. Cochrane Database Syst Rev. 2009;2009(4):CD004977. doi: 10.1002/14651858.CD004977.pub2 19821336 PMC6464899

[46] Kieninger MP, Caballero EG, Sosa AA, Amarilla CT, Jáuregui B, Janusz CB, et al. Cost-effectiveness analysis of pneumococcal conjugate vaccine introduction in Paraguay. Vaccine. 2015;33 Suppl 1:A143-53. doi: 10.1016/j.vaccine.2014.12.078 25919155

[47] Barnabas RV, Brown ER, Onono MA, Bukusi EA, Njoroge B, Winer RL, et al. Efficacy of single-dose HPV vaccination among young African women. NEJM Evid. 2022;1(5):EVIDoa2100056. doi: 10.1056/EVIDoa2100056 35693874 PMC9172784

[48] Parkin DM, Louie KS, Clifford G. Burden and trends of type-specific human papillomavirus infections and related diseases in the Asia Pacific region. Vaccine. 2008;26 Suppl 12:M1-16. doi: 10.1016/j.vaccine.2008.05.010 18945410

[49] Kjaer SK, Nygård M, Sundström K, Dillner J, Tryggvadottir L, Munk C, et al. Final analysis of a 14-year long-term follow-up study of the effectiveness and immunogenicity of the quadrivalent human papillomavirus vaccine in women from four nordic countries. EClinicalMedicine. 2020;23:100401. doi: 10.1016/j.eclinm.2020.100401 32637895 PMC7329692

[50] Talbird SE, Taylor TN, Knoll S, Frostad CR, García Martí S. Outcomes and costs associated with PHiD-CV, a new protein D conjugate pneumococcal vaccine, in four countries. Vaccine. 2010;28 Suppl 6:G23-9. doi: 10.1016/j.vaccine.2010.06.016 21075266

[51] Rogawski ET, Platts-Mills JA, Colgate ER, Haque R, Zaman K, Petri WA, et al. Quantifying the impact of natural immunity on rotavirus vaccine efficacy estimates: a clinical trial in Dhaka, Bangladesh (PROVIDE) and a simulation study. J Infect Dis. 2018;217(6):861–8. doi: 10.1093/infdis/jix668 29514306 PMC5853827

[52] Clark A, van Zandvoort K, Flasche S, Sanderson C, Bines J, Tate J, et al. Efficacy of live oral rotavirus vaccines by duration of follow-up: a meta-regression of randomised controlled trials. Lancet Infect Dis. 2019;19(7):717–27. doi: 10.1016/S1473-3099(19)30126-4 31178289 PMC6595176

[53] WHO and UNICEF estimates of immunization coverage: 2021revision [Internet]; 2022. Available from: https://www.who.int/teams/immunization-vaccines-and-biologicals/immunization-analysis-and-insights/global-monitoring/immunization-coverage/who-unicef-estimates-of-national-immunization-coverage

[54] Varghese T, Kang G, Steele AD. Understanding rotavirus vaccine efficacy and effectiveness in countries with high child mortality. Vaccines (Basel). 2022;10(3):346. doi: 10.3390/vaccines10030346 35334978 PMC8948967

[55] Zaman K, Schuind AE, Adjei S, Antony K, Aponte JJ, Buabeng PBY, et al. Safety and immunogenicity of Innovax bivalent human papillomavirus vaccine in girls 9–14 years of age: Interim analysis from a phase 3 clinical trial. Vaccine. 2024;42(9):2290–8. doi: 10.1016/j.vaccine.2024.02.077 38431444 PMC11007388

[56] Market Information for Access (MI4A) vaccine purchase database [Internet]; 2024 [cited 23 Aug 2024]. Available from: https://www.who.int/teams/immunization-vaccines-and-biologicals/vaccine-access/mi4a/mi4a-vaccine-purchase-data

[57] Portnoy A, Vaughan K, Clarke-Deelder E, Suharlim C, Resch SC, Brenzel L, et al. Producing standardized country-level immunization delivery unit cost estimates. Pharmacoeconomics. 2020;38(9):995–1005. doi: 10.1007/s40273-020-00930-6 32596785 PMC7437655

[58] Immunization Costing Action Network (ICAN). Immunization Delivery Cost Catalogue. Washington; 2019.

[59] Immunization Costing Action Network (ICAN). Immunization delivery cost catalogue. Washington: ThinkWell; 2019.

[60] Labour force participation rate, female [Internet]. World Bank data. [cited 22 Dec 2017]. Available from: https://data.worldbank.org/indicator/SL.TLF.CACT.FE.ZS

[61] 2019 GDP per capita in current US$: World Bank Data. [cited 22 Dec 2021]. Available from: https://data.worldbank.org/indicator/NY.GDP.PCAP.CD

[62] Hutubessy R, Chisholm D, Edejer TT-T. Generalized cost-effectiveness analysis for national-level priority-setting in the health sector. Cost Eff Resour Alloc. 2003;1(1):8. doi: 10.1186/1478-7547-1-8 14687420 PMC320499

[63] Pichon-Riviere A, Drummond M, Palacios A, Garcia-Marti S, Augustovski F. Determining the efficiency path to universal health coverage: cost-effectiveness thresholds for 174 countries based on growth in life expectancy and health expenditures. Lancet Glob Health. 2023;11(6):e833–42. doi: 10.1016/S2214-109X(23)00162-6 37202020

[64] Woods B, Revill P, Sculpher M, Claxton K. Country-level cost-effectiveness thresholds: initial estimates and the need for further research. Value Health. 2016;19(8):929–35. doi: 10.1016/j.jval.2016.02.017 27987642 PMC5193154

[65] World Health Organization. WHO guide for standardization of economic evaluations of immunization programmes. Geneva: World Health Organization; 2019.

[66] Clark A, Mahmud S, Debellut F, Pecenka C, Jit M, Perin J, et al. Estimating the global impact of rotavirus vaccines on child mortality. Int J Infect Dis. 2023;137:90–7. doi: 10.1016/j.ijid.2023.10.005 37863311 PMC10689250

[67] Davis R. Teaching note—teaching project simulation in excel using PERT-beta distributions. INFORMS Trans Educ. 2008;8(3):139–48. doi: 10.1287/ited.1080.0013

[68] Bertram MY, Lauer JA, De Joncheere K, Edejer T, Hutubessy R, Kieny M-P, et al. Cost-effectiveness thresholds: pros and cons. Bull World Health Organ. 2016;94(12):925–30. doi: 10.2471/BLT.15.164418 27994285 PMC5153921

[69] Gavi. Gavi’s approach to engaging with middle-income countries 2023 [updated 13/3/2023; cited 2024 16/2/2024]. Available from: https://www.gavi.org/types-support/sustainability/gavi-mics-approach#:~:text=In%20this%20context%2C%20the%20MICs,select%20never%2DGavi%20eligible%20countries

[70] Russell FM, Bowen A, Cotton M, Mascareñas A, O’Ryan M, World Society of Pediatric Infectious Diseases. World Society for Pediatric Infectious Diseases calls for action to ensure fair prices for vaccines. Lancet Glob Health. 2024;12(1):e22–4. doi: 10.1016/S2214-109X(23)00457-6 37980913

[71] Li X, Bilcke J, Asare EO, Wenger C, Kwon J, Bont L, et al. Cost per episode of diarrhea and respiratory syncytial virus (RSV) in 128 low- and middle-income countries: how well do disease-specific and WHO-CHOICE estimates align?. medRxiv. 2024:2024.07.17.24310217. doi: 10.1101/2024.07.17.24310217 40850784 PMC12382583

[72] Bertran M, D’Aeth JC, Abdullahi F, Eletu S, Andrews NJ, Ramsay ME, et al. Invasive pneumococcal disease 3 years after introduction of a reduced 1 + 1 infant 13-valent pneumococcal conjugate vaccine immunisation schedule in England: a prospective national observational surveillance study. Lancet Infect Dis. 2024;24(5):546–56. doi: 10.1016/S1473-3099(23)00706-5 38310905

[73] Russell FM, Chokephaibulkit K. Will two doses of pneumococcal conjugate vaccine be enough?. Lancet Infect Dis. 2024;24(5):449–51. doi: 10.1016/S1473-3099(23)00812-5 38310907

[74] Hutubessy R, Lauer JA, Giersing B, Sim SY, Jit M, Kaslow D, et al. The Full Value of Vaccine Assessments (FVVA): a framework for assessing and communicating the value of vaccines for investment and introduction decision-making. BMC Med. 2023;21(1). doi: 10.1186/s12916-023-02929-0 PMC10318807 37400797

[75] Park J, Goldstein J, Haran M, Ferrari M. An ensemble approach to predicting the impact of vaccination on rotavirus disease in Niger. Vaccine. 2017;35(43):5835–41. doi: 10.1016/j.vaccine.2017.09.020 28941619 PMC7185385

[76] Jit M, Brisson M, Portnoy A, Hutubessy R. Cost-effectiveness of female human papillomavirus vaccination in 179 countries: a PRIME modelling study. Lancet Glob Health. 2014;2(7):e406-14. doi: 10.1016/S2214-109X(14)70237-2 25103394

[77] Pitzer VE, Atkins KE, de Blasio BF, Van Effelterre T, Atchison CJ, Harris JP, et al. Direct and indirect effects of rotavirus vaccination: comparing predictions from transmission dynamic models. PLoS One. 2012;7(8):e42320. doi: 10.1371/journal.pone.0042320 22912699 PMC3418263

[78] Rose J, Homa L, Meropol SB, Debanne SM, Bielefeld R, Hoyen C, et al. Health impact and cost-effectiveness of a domestically-produced rotavirus vaccine in India: a model based analysis. PLoS One. 2017;12(11):e0187446. doi: 10.1371/journal.pone.0187446 29099848 PMC5669435

[79] Reyburn R, Tuivaga EJ, Ratu FT, Dunne EM, Nand D, Kado J, et al. The impact of 10-valent pneumococcal vaccine introduction on invasive disease in Fiji. Lancet Reg Health West Pac. 2022;20:100352. doi: 10.1016/j.lanwpc.2021.100352 35028629 PMC8741523

[80] Shiri T, Datta S, Madan J, Tsertsvadze A, Royle P, Keeling MJ, et al. Indirect effects of childhood pneumococcal conjugate vaccination on invasive pneumococcal disease: a systematic review and meta-analysis. Lancet Glob Health. 2017;5(1):e51–9. doi: 10.1016/S2214-109X(16)30306-0 27955789

[81] Chan J, Mungun T, Batsaixan P, Ulziibayar M, Suuri B, Otgonbayar D, et al. Direct and indirect effects of 13-valent pneumococcal conjugate vaccine on pneumococcal carriage in children hospitalised with pneumonia from formal and informal settlements in Mongolia: an observational study. Lancet Reg Health West Pac. 2021;15:100231. doi: 10.1016/j.lanwpc.2021.100231 34528012 PMC8342962

[82] Chen C, Ang G, Akksilp K, Koh J, Scott JAG, Clark A, et al. Re-evaluating the impact and cost-effectiveness of pneumococcal conjugate vaccine introduction in 112 low-income and middle-income countries in children younger than 5 years: a modelling study. Lancet Glob Health. 2024;12(9):e1485–97. doi: 10.1016/S2214-109X(24)00232-8 39151983 PMC11345449

