# Supplementary material for: Evaluation of rotavirus, pneumococcal conjugate and human papillomavirus vaccination in four Pacific island countries: A cost-effectiveness modelling study
Source: PLoS Med. 2026 Feb 12;23(2):e1004604. doi: 10.1371/journal.pmed.1004604 (PMC12900362; doi:10.1371/journal.pmed.1004604)
Supplement: S1 Text — (DOCX) [file pmed.1004604.s001.docx]

**S1 Text**

**Country context**

Pacific Island Countries typically consist of several land masses stretching across large areas of the Pacific Ocean. Populations of these countries are relatively small compared to other areas in the world with much of the population residing in rural areas. Wide dispersion of populations across islands and remote locations leads to challenges in health service delivery and logistics.

All four countries face a high burden of non-communicable disease; however, respiratory diseases continue to contribute significantly to the burden of mortality. Under five mortality rates are below those reported for other small island developing states (37 deaths per 1,000 live births) and closer to Fiji (26.8 deaths). In all countries, and in particular Tuvalu, small populations make under five mortality rates highly sensitive to even small changes in absolute numbers of deaths.

Unique geographic features of these countries make them highly susceptible to impacts of environmental, economic and health crises. This susceptibility is captured in the Multidimensional Vulnerability Index (MVI) - an indicator quantifying a country’s structural vulnerability and lack of resilience, with high scores indicating high vulnerability to shocks and lack of resilience. The MVI scores for all four countries, which range from 54 to 64, are above those of other middle-income countries (MVI of 49).

**Table 1 Summary of key information on countries included in the study.** Fiji has been included for context given that data has been drawn from Fiji where country-specific data were not available.

|  | **Fiji** | **Samoa** | **Tonga** | **Tuvalu** | **Vanuatu** |
| --- | --- | --- | --- | --- | --- |
| Population (2023)^a^ | 924,145 | 216,663 | 104,597 | 9,816 | 320,409 |
| Rural population (% of total population) ^a^ | 41% | 82% | 77% | 34% | 74% |
| Fertility rate, total (births per woman; 2022) ^a^ | 2.5 | 3.9 | 3.2 | 3.1 | 3.7 |
| Multidimensional Vulnerability Index (2024)^b^ | 51.7 | 62.5 | 57.0 | 64.3 | 54.4 |
| Out of pocket expenditure as % CHE (2021)^c^ | 17.85% | 11.17% | 3.49% | 0.36% | 7.21% |
| External health expenditure as % CHE (2021) ^c^ | 9.73% | 12.89% | 46.13% | 47.68% | 63.03% |
| Domestic government health expenditure as % CHE (2021) ^c^ | 62.38% | 75.30% | 47.44% | 51.90% | 27.10% |
| Life expectancy at birth, total (males, females) (2022) ^a^ | 68  (66,70) | 73  (70, 75) | 71  (69,74) | 65  (61,69) | 70  (68,73) |
| Under-5 mortality (per 1,000 livebirths; 2020)^d^ | 26.8 | 17.1 | 11.5 | 21.7 | 19.2 |

CHE = current health expenditure

^a^ United Nations Population Division. World Population Prospects: 2024 Revision; ^b^ United Nations President of the General Assembly’s High Level Panel on the Development of a Multidimensional Vulnerability Index; ^c^ WHO Global Expenditures Database; ^d^ UNICEF Data Child Health Statistics
