# Supplementary material for: Evaluation of rotavirus, pneumococcal conjugate and human papillomavirus vaccination in four Pacific island countries: A cost-effectiveness modelling study
Source: PLoS Med. 2026 Feb 12;23(2):e1004604. doi: 10.1371/journal.pmed.1004604 (PMC12900362; doi:10.1371/journal.pmed.1004604)
Supplement: S2 Text — (DOCX) [file pmed.1004604.s002.docx]

**S2 Text**

**UNIVAC model description**

*Overview*

UNIVAC is an Excel-based proportionate outcomes model that calculates incremental cost-effectiveness ratios (ICERs) and other indicators, including numbers of prevented cases, outpatient visits, admissions and deaths. Estimates of costs, health benefits and cost-effectiveness are calculated by tracking the experience of annual birth cohorts to age 5 years for Pneumococcal conjugate vaccines (PCV) and rotavirus vaccine (RVV), and annual cohorts of girls aged ten throughout a lifetime horizon for human papillomavirus vaccine (HPVV). See Figure 1 for a simplified schematic of the model. Further detail on the structure of the model is available elsewhere and briefly summarised below.[1]


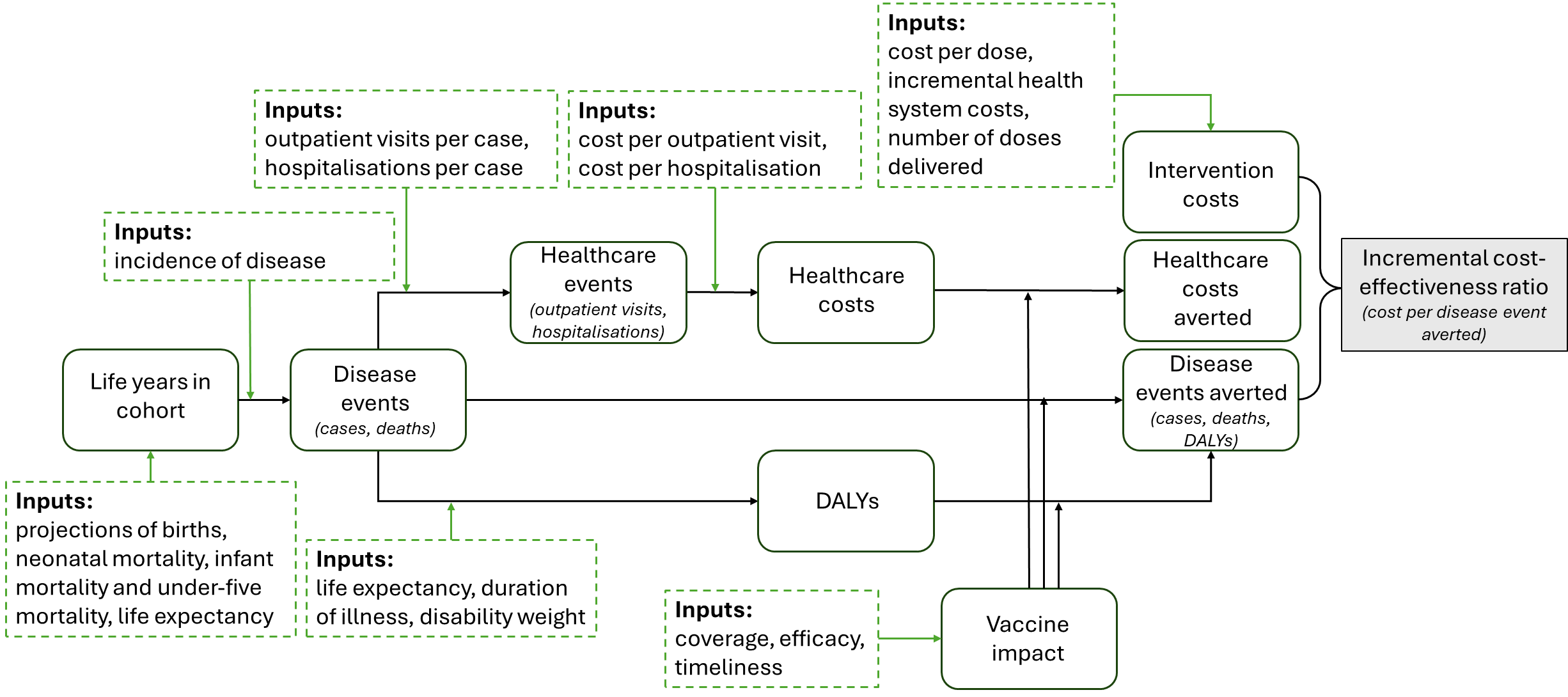


**Figure 1: Simplified schematic of UNIVAC model structure.** DALY= disability-adjusted life year.

*Disease incidence, years of life lost and disability-adjusted life years.*

For PCV and RVV, life-years lived between birth and age 5.0 years are derived for each birth cohort from 2021 - 2031 using UN World Population Prospects (UNWPP) annual projections of the number of individuals alive in each single age of life and single calendar year over time. For HPVV, we track successive cohorts of ten-year old females (corresponding to birth cohorts 2011-2021), and use UNWPP estimates the number of females expected to be alive in each single age of life and single calendar year over the lifetime of each cohort.

Life-years between birth and age 5.0 years are multiplied by rates of disease cases and deaths (per 100,000) to estimate numbers of cases and deaths expected to occur without vaccination between birth and age five years for PCV and RVV. Age distributions of rotavirus and pneumococcal disease in children under 5 years (by week of age) were based on analysis of inpatient datasets for each disease, with the same distribution applied to severe and non-severe disease [2, 3]. For HPV, rates of cervical cancer (per 100,000) are applied to the number of females expected to be alive in each 5-year age group over the lifetime of the cohort.

Historical time-series estimates of pneumonia and diarrhoea deaths have declined in the absence of vaccination.[4] To avoid over-stating the impact of PCV and RVV, the model assumes the rotavirus and pneumococcal mortality rate will decrease without vaccination at the same rate as the overall under-five mortality rate. No decline in the incidence of disease cases is assumed, so case fatality ratios are assumed to decline in each successive year in the absence of vaccination. There is insufficient data to inform plausible assumptions about how cervical cancer rates will change over the coming decades and as such, cancer incidence inputs were kept constant in the model over the time horizon modelled.

Current and projected life expectancy estimates by age and year are used to calculate years of life lost (YLL) due to premature mortality from the age/year of disease to death. Years of life with disease (YLDs) are calculated by multiplying disability weights by the average duration of illness. YLLs and YLDs are summed to give disability-adjusted life years DALYs attributed to the year of disease onset.

*Healthcare utilisation and costs*

For PCV and RVV, healthcare utilisation and costs are calculated from the onset of disease through to the age of five, except for pneumococcal meningitis sequelae, where healthcare costs are calculated over a lifetime horizon. For HPVV, healthcare utilisation and costs are calculated from the onset of disease throughout a lifetime horizon. As repeat contacts with the health system are common for cervical cancer treatment, the ‘hospitalisation’ category captures the cost of all relevant contacts with the health system. For simplicity all costs are assumed to occur in the first year of cancer diagnosis.

The numbers of outpatient visits and hospitalisations are estimated multiplying number of cases by an assumed average number of visits and admissions per case of each disease. See Table 1 for summary of disease and healthcare utilisation events modelled for each condition.

**Table 1: Summary of disease and healthcare utilisation events modelled for each condition.**

|  | **Disease events** | | **Healthcare utilisation events** | |
| --- | --- | --- | --- | --- |
|  | *Cases* | *Deaths* | *Outpatient visits* | *Hospitalisations* |
| **Cervical Cancer** | | | | |
| Local cases | ✓ | ✓ |  | ✓ |
| Regional cases | ✓ | ✓ |  | ✓ |
| Distant cases | ✓ | ✓ |  | ✓ |
| **Pneumococcal disease** | | | | |
| Acute otitis media | ✓ |  | ✓ |  |
| Pneumonia (non-severe) | ✓ |  | ✓ |  |
| Pneumonia (severe) | ✓ | ✓ | ✓ | ✓ |
| Meningitis | ✓ | ✓ | ✓ | ✓ |
| NPNM | ✓ | ✓ | ✓ | ✓ |
| Meningitis sequelae | ✓ |  | ✓ |  |
| **Rotavirus disease** | | | | |
| Non-severe RVGE | ✓ |  | ✓ |  |
| Severe RVGE | ✓ | ✓ | ✓ | ✓ |
| Intussusception | ✓ | ✓ |  | ✓ |

NPNM: non-pneumonia non-meningitis invasive diseases; RVGE: Rotavirus gastroenteritis

Costs per outpatient visit and hospitalisation are estimated separately for governments and households as outlined in S5 Appendix. Annual healthcare costs (for each payer) are calculated by multiplying the cost per healthcare event (outpatient visit or hospitalisation) by the number of events in each year. Future healthcare costs are discounted at 3% (6% in a sensitivity analysis) and total healthcare costs are calculated by summing annual discounted outpatient visit costs and hospitalisation costs. For the budget impact analysis, costs are not discounted.

*Vaccination program costs*

Vaccination program costs include the costs of the vaccine dose (including freight, handling, wastage and vaccine supplies) and the incremental costs to the health system of delivering an additional vaccine in the existing immunisation program (including cold-chain logistics, training, community mobilisation, monitoring and evaluation etc). Vaccination programme costs are assumed to occur in the first year of each cohort.

The cost per dose of vaccine administered is calculated by inflating the vaccine price per dose by multipliers representing freight and handling costs (both expressed as a % of price per dose). This cost is further inflated to reflect wastage rates (expressed as a % of doses). The same process is applied to estimate the cost of syringes and safety boxes (expressed as a cost per dose). Dose costs and other supply costs are then combined to estimate the final vaccine cost per dose delivered.

In addition to estimating the cost of vaccine doses and supplies, it is important to capture the incremental health system costs of vaccine introduction and recurrent delivery (expressed as a cost per dose delivered). This cost was derived from the literature.[5]

To estimate the total numbers of vaccines administered, the model takes into account the differences in coverage for each dose. Coverage estimates are applied to the number of target infants/females alive at the mid-year population of each single calendar year of interest.

Total program costs are calculated by summing the vaccine dose cost and the incremental health system costs (both expressed as a cost per dose delivered) and multiplying by the total numbers of vaccine doses administered in each year, with future costs discounted at 3% (6% in a sensitivity analysis). For the budget impact analysis, costs are not discounted.

*Vaccine impact*

For PCV and RVV, estimates of vaccination impact are restricted to children aged under five years of age. The impact is calculated by multiplying the expected number of disease and healthcare events (cases, clinic visits, hospitalisations, deaths) in each week of age up to five years by the expected coverage of vaccination in each week of age (adjusted for realistic vaccine delays/timeliness) and the expected efficacy of vaccination in each week of age (adjusted for the waning vaccine protection).

For HPVV, vaccination impact is calculated for the target cohort (taking into account vaccine coverage, the cervical cancer type distribution, and the efficacy of the vaccine against each type), and is assumed to provide lifetime protection.

DALYs averted are discounted at 3% per year (0% - 6% in the sensitivity analysis).

*Model assumptions and limitations*

UNIVAC is not a transmission dynamic model and thus excludes indirect effects (both positive and negative). More detailed validation against real-world post-introduction evidence of impact is needed to understand the direction and magnitude of these effects in Samoa, Tonga, Tuvalu and Vanuatu. However, the available data in these countries are insufficient to allow validation of modelled estimates (against real-world estimates of post-introduction vaccine impact) and/or parameterisation of a country-specific transmission dynamic model.

For simplicity, the parameters are assumed to be independent and were sampled from PERT-Beta distributions. For each parameter, the best available central input estimate represented the mean of the distribution and the low and high input estimates represented the range.

# References

1. Clark A, Jauregui B, Griffiths U, Janusz CB, Bolaños-Sierra B, Hajjeh R, et al. TRIVAC decision-support model for evaluating the cost-effectiveness of Haemophilus influenzae type b, pneumococcal and rotavirus vaccination. Vaccine. 2013;31:C19-C29. doi: <https://doi.org/10.1016/j.vaccine.2013.05.045>.

2. Hasso-Agopsowicz M, Ladva CN, Lopman B, Sanderson C, Cohen AL, Tate JE, et al. Global Review of the Age Distribution of Rotavirus Disease in Children Aged <5 Years Before the Introduction of Rotavirus Vaccination. Clin Infect Dis. 2019;69(6):1071-8. doi: 10.1093/cid/ciz060. PubMed PMID: 30689799; PubMed Central PMCID: PMCPMC6736387.

3. Russell F, Sanderson C, Temple B, Mulholland EK. Global review of the distribution of pneumococcal disease by age and region2011. Available from: <https://www.who.int/immunization/sage/6_Russel_review_age_specific_epidemiology_PCV_schedules_session_nov11.pdf>.

4. Perin J, Mulick A, Yeung D, Villavicencio F, Lopez G, Strong KL, et al. Global, regional, and national causes of under-5 mortality in 2000-19: an updated systematic analysis with implications for the Sustainable Development Goals. Lancet Child Adolesc Health. 2022;6(2):106-15. Epub 20211117. doi: 10.1016/S2352-4642(21)00311-4. PubMed PMID: 34800370; PubMed Central PMCID: PMCPMC8786667.

5. Portnoy A, Vaughan K, Clarke-Deelder E, Suharlim C, Resch SC, Brenzel L, Menzies NA. Producing Standardized Country-Level Immunization Delivery Unit Cost Estimates. Pharmacoeconomics. 2020;38(9):995-1005. doi: 10.1007/s40273-020-00930-6. PubMed PMID: 32596785; PubMed Central PMCID: PMCPMC7437655.
