# Supplementary material for: Evaluation of rotavirus, pneumococcal conjugate and human papillomavirus vaccination in four Pacific island countries: A cost-effectiveness modelling study
Source: PLoS Med. 2026 Feb 12;23(2):e1004604. doi: 10.1371/journal.pmed.1004604 (PMC12900362; doi:10.1371/journal.pmed.1004604)
Supplement: S1 Appendix — (DOCX) [file pmed.1004604.s004.docx]

**S1 Appendix**

**Input parameters for estimating disease burden**

**Table A: Demographic input parameters.**

|  | **Samoa** | **Tonga** | **Tuvalu** | **Vanuatu** | **Source/notes** |
| --- | --- | --- | --- | --- | --- |
| **Birth cohort (all sexes, 2021)^a^** | 4,621 | 2,509 | 260 | 7,830 | UN Population projections^c^ |
| **10-year old girls (2021)** | 2,335 | 1,251 | 129 | 3,233 |  |
| **Future year cohorts to 2030** | Year specific | | | |  |
| **Life expectancy at birth – both sexes (2021)^b^** | 73 years | 71 years | 71 years | 70 years |  |
| **Life expectancy at birth females (2011)^b^** | 74 years | 73 years | 73 years | 71 years |  |

^a^ Birth cohort number reflects males and females (0-1 years) for the year

^b^ Life expectancy is modelled as changing over time based on UN Population Projections for each of the countries.

^c^ Tuvalu demographic projections were not available from UN Population Projections data; demographic data was based on Tonga’s population data, scaled to 10%.

**Table B: Inputs for cervical cancer epidemiology.**

|  | **Base Case** | **Low** | **High** | **Source/notes** |
| --- | --- | --- | --- | --- |
| **Cases (per 100,000 per year)^a^** | | | | |
| 10-15 yrs | 0 | 0 | 0 | Base case: Assumed same as well-documented age-specific incidence of cervical cancer in Melanesian Fijian women [1].  Low: Scaled down by 0.31 to reflect an estimate of incidence from Tonga [2].  High: As for base case, with last observed incidence projected forward for older age groups. |
| 15-20 yrs | 0 | 0 | 0 |  |
| 20-25 yrs | 1·6 | 0·5 | 1·6 |  |
| 25-30 yrs | 18·6 | 5·8 | 18·6 |  |
| 30-35 yrs | 34·0 | 10·5 | 34·0 |  |
| 35-40 yrs | 86·2 | 26·7 | 86·2 |  |
| 40-45 yrs | 87·2 | 27·0 | 87·2 |  |
| 45-50 yrs | 96·7 | 29·9 | 96·7 |  |
| 50-55 yrs | 131·2 | 40·6 | 131·2 |  |
| 55-60 yrs | 171·0 | 53·0 | 171·0 |  |
| 60-65 yrs | 104·3 | 32·3 | 104·3 |  |
| 65-70 yrs | 164·9 | 51·1 | 164·9 |  |
| 70-75 yrs | 0 | 0 | 164·9 |  |
| 75-80 yrs | 0 | 0 | 164·9 |  |
| 80-85 yrs | 0 | 0 | 164·9 |  |
| 85-90 yrs | 0 | 0 | 164·9 |  |
| 90-95 yrs | 0 | 0 | 164·9 |  |
| 95-99 yrs | 0 | 0 | 164·9 |  |
| **Deaths (per 100,000 per year)** | | | | |
| 10-15 yrs | 0 | 0 | 0 | Base case: Assumed same as well-documented age-specific incidence of cervical cancer deaths in Melanesian Fijian women [1].  Low: Scaled down to reflect an estimate of incidence from Tonga [2].  High: As for base case, with last observed incidence projected forward for older age groups. |
| 15-20 yrs | 0 | 0 | 0 |  |
| 20-25 yrs | 1·0 | 0·3 | 1 |  |
| 25-30 yrs | 3·3 | 1·0 | 3·3 |  |
| 30-35 yrs | 10·0 | 3·1 | 10 |  |
| 35-40 yrs | 17·1 | 5·3 | 17·1 |  |
| 40-45 yrs | 42·7 | 13·2 | 42·7 |  |
| 45-50 yrs | 52·0 | 16·1 | 52 |  |
| 50-55 yrs | 85·7 | 26·5 | 85·7 |  |
| 55-60 yrs | 73·2 | 22·7 | 73·2 |  |
| 60-65 yrs | 72·2 | 22·4 | 72·2 |  |
| 65-70 yrs | 171·9 | 53·2 | 171·9 |  |
| 70-75 yrs | 0 | 0 | 171·9 |  |
| 75-80 yrs | 0 | 0 | 171·9 |  |
| 80-85 yrs | 0 | 0 | 171·9 |  |
| 85-90 yrs | 0 | 0 | 171·9 |  |
| 90-95 yrs | 0 | 0 | 171·9 |  |
| 95-99 yrs | 0 | 0 | 171·9 |  |

^a^ The age specific incidence of cervical cancer was further broken down into an assumed distribution across severity grades. The assumption for all countries was: 19% local, 73% regional, 9% distant assuming no screening available in country, based on Campos N et al. [3]. A calculation within UNIVAC, based on all information about staging of cervical cancer cases in low and low-middle income countries in GLOBOCAN 2012[4].

**Table C: Age distribution of pneumococcal and rotavirus disease under 5 years of age**

|  | **Samoa** | **Tonga** | **Tuvalu** | **Vanuatu** | **Source/notes** |
| --- | --- | --- | --- | --- | --- |
| **Pneumococcal disease event age distribution** |  |  |  |  | From a global review of age- and region-specific rates of pneumococcal disease [5]. |
| *By 1 month of age* | 0% | 0% | 0% | 0% |  |
| *By 2 months of age* | 0% | 0% | 0% | 0% |  |
| *By 3 months of age* | 1% | 1% | 1% | 1% |  |
| *By 6 months of age* | 5% | 5% | 5% | 5% |  |
| *By 1 year of age* | 27% | 27% | 27% | 27% |  |
| *By 2 years of age* | 75% | 75% | 75% | 75% |  |
| *By 3 years of age* | 92% | 92% | 92% | 92% |  |
| *By 4 years of age* | 98% | 98% | 98% | 98% |  |
| *By 5 years of age* | 100% | 100% | 100% | 100% |  |
| **Rotavirus disease event age distribution** |  |  |  |  | From a global review of age- and region-specific rates of rotavirus disease [6]. |
| *By 1 month of age* | 0% | 0% | 0% | 0% |  |
| *By 2 months of age* | 1% | 1% | 1% | 1% |  |
| *By 3 months of age* | 4% | 4% | 4% | 4% |  |
| *By 6 months of age* | 20% | 20% | 20% | 21% |  |
| *By 1 year of age* | 58% | 58% | 58% | 63% |  |
| *By 2 years of age* | 89% | 89% | 89% | 92% |  |
| *By 3 years of age* | 97% | 97% | 97% | 98% |  |
| *By 4 years of age* | 99% | 99% | 99% | 99% |  |
| *By 5 years of age* | 100% | 100% | 100% | 100% |  |
| **Intussusception age distribution** |  |  |  |  | From a systematic review of the age-specific incidence of intussusception [7]. |
| *By 2 months of age* | 0% | 0% | 0% | 0% |  |
| *By 3 months of age* | 2% | 2% | 2% | 2% |  |
| *By 6 months of age* | 10% | 10% | 10% | 10% |  |
| *By 1 year of age* | 39% | 39% | 39% | 39% |  |
| *By 2 years of age* | 75% | 75% | 75% | 75% |  |
| *By 3 years of age* | 90% | 90% | 90% | 90% |  |
| *By 4 years of age* | 96% | 96% | 96% | 96% |  |
| *By 5 years of age* | 100% | 100% | 100% | 100% |  |

**Table D: Inputs for modelling disability adjusted life years. Same disability weights and durations used for all countries.**

|  | **Base Case** | **Low** | **High** | **Source/notes** |
| --- | --- | --- | --- | --- |
| **Disability weight^a^** |  |  |  |  |
| **Cervical cancer^b^** |  |  |  |  |
| Local | 0·288 | 0·193 | 0·399 | proxy: Diagnosis and primary therapy phase of cervical cancer |
| Regional | 0·451 | 0·307 | 0·600 | proxy: Metastatic phase of cervical cancer |
| Distant | 0·540 | 0·377 | 0·687 | proxy: Terminal phase of cervical cancer |
| **Pneumococcal disease** |  |  |  |  |
| Sp acute otitis media | 0·013 | 0·007 | 0·024 |  |
| Sp pneumonia (non-severe) | 0·051 | 0·032 | 0·074 | proxy: moderate lower respiratory infection |
| Sp pneumonia (severe) | 0·133 | 0·0885 | 0·19 | proxy: severe lower respiratory infection |
| Sp meningitis | 0·133 | 0·088 | 0·19 | Assumption (same as severe pneumonia) |
| Sp NPNM | 0·133 | 0·088 | 0·19 | Assumption (same as meningitis) |
| Sp meningitis sequelae | 0·26 | 0·153 | 0·364 | Assumption based on UNIVAC defaults |
| **Rotavirus disease** |  |  |  |  |
| Non-severe RVGE | 0·188 | 0·125 | 0·264 | proxy: moderate diarrhoea |
| Severe RVGE | 0·247 | 0·164 | 0·348 | proxy: severe diarrhoea |
| Intussusception | 0·324 | 0·22 | 0·442 | proxy: abdominopelvic problem, severe |
| **Average duration of illness (time spent living with disease in years)** | | | | |
| **Cervical cancer** |  |  |  |  |
| Local | 0·4 | 0·4 | 10 | Base case/low: [8]; High: [9] |
| Regional | 0·768 | 0·768 | 7·5 | Base case/low: [8]; High: [9] |
| Distant | 0·083 | 0·083 | 2 | Base case/low: [8]; High: [9] |
| **Pneumococcal disease** |  |  |  |  |
| Sp acute otitis media | 0·02 | 0·02 | 0·02 | Assumption |
| Sp pneumonia (non-severe) | 0·02 | 0·02 | 0·02 | Assumption |
| Sp pneumonia (severe) | 0·03 | 0·02 | 0·06 | Assumption |
| Sp meningitis | 0·03 | 0·02 | 0·06 | Assumption |
| Sp NPNM | 0·03 | 0·02 | 0·06 | Assumption |
| Sp meningitis sequelae | 50 | 50 | 50 | Assumption |
| **Rotavirus disease** |  |  |  |  |
| Non-severe RVGE | 0·008 | 0·008 | 0·008 | Center for Disease Control[10] |
| Severe RVGE | 0·019 | 0·019 | 0·019 | Center for Disease Control[10] |
| Intussusception | 0·019 | 0·019 | 0·058 | Assumption |

NPNM: non-pneumonia non-meningitis invasive diseases; RVGE: Rotavirus gastroenteritis

^a^ GBD 2017 study[8] for all diseases, proxy provided where disease not exact match to GBD 2017. Death valued at disability weight of 1 for all diseases.

^b^ Disability weights for early stage cancer (local/regional) are only applied to the estimated proportion of women with disease who are diagnosed, based on country-specific opportunistic screening rates. Calculated as: number of pap smears conducted in 10-year period (based on most recent local data available scaled up to 10 years) / number of women aged 20 years and up (UN Population Projections) to get proportion of women who would get at least 1 pap smear in 10 years. Multiplied by 55% pap smear sensitivity to get proportion of cases that would be diagnosed and hospitalized. In Samoa, account for data available of 18% unsatisfactory pap smear results. In Tuvalu, assume 10% of pap smears are repeats due to up to 1 year delay for results to be available (samples sent to Fiji).

# References

1. Law I, Fong JJ, Buadromo EM, Samuela J, Patel MS, Garland SM, et al. The high burden of cervical cancer in Fiji, 2004-07. Sex Health. 2013;10(2):171-8. Epub 2013/04/06. doi: 10.1071/SH12135. PubMed PMID: 23557630.

2. Foliaki S, Best D, Akau'ola S, Cheng S, Borman B, Pearce N. Cancer incidence in four pacific countries: Tonga, Fiji Islands, Cook Islands and Niue. Pac Health Dialog. 2011;17(1):21-32. Epub 2011/03/01. PubMed PMID: 23008968.

3. Campos NG, Sharma M, Clark A, Lee K, Geng F, Regan C, et al. The health and economic impact of scaling cervical cancer prevention in 50 low- and lower-middle-income countries. Int J Gynaecol Obstet. 2017;138 Suppl 1:47-56. Epub 2017/07/12. doi: 10.1002/ijgo.12184. PubMed PMID: 28691334.

4. Torre LA, Bray F, Siegel RL, Ferlay J, Lortet-Tieulent J, Jemal A. Global cancer statistics, 2012. CA Cancer J Clin. 2015;65(2):87-108. Epub 20150204. doi: 10.3322/caac.21262. PubMed PMID: 25651787.

5. Russell FS, C. Temple, B. Mulholland, E.K. Global review of the distribution of pneumococcal disease by age and region. World Health Organization,, 2011.

6. Hasso-Agopsowicz M, Ladva CN, Lopman B, Sanderson C, Cohen AL, Tate JE, et al. Global Review of the Age Distribution of Rotavirus Disease in Children Aged <5 Years Before the Introduction of Rotavirus Vaccination. Clin Infect Dis. 2019;69(6):1071-8. doi: 10.1093/cid/ciz060. PubMed PMID: 30689799; PubMed Central PMCID: PMCPMC6736387.

7. Clark AD, Hasso-Agopsowicz M, Kraus MW, Stockdale LK, Sanderson CFB, Parashar UD, Tate JE. Update on the global epidemiology of intussusception: a systematic review of incidence rates, age distributions and case-fatality ratios among children aged <5 years, before the introduction of rotavirus vaccination. Int J Epidemiol. 2019;48(4):1316-26. Epub 2019/03/18. doi: 10.1093/ije/dyz028. PubMed PMID: 30879038; PubMed Central PMCID: PMCPMC6693807.

8. GBD 2017 Disease and Injury Incidence and Prevalence Collaborators. Global, regional, and national incidence, prevalence, and years lived with disability for 354 diseases and injuries for 195 countries and territories, 1990-2017: a systematic analysis for the Global Burden of Disease Study 2017. Lancet. 2018;392(10159):1789-858. Epub 20181108. doi: 10.1016/s0140-6736(18)32279-7. PubMed PMID: 30496104; PubMed Central PMCID: PMCPMC6227754.

9. Anwari P, Debellut F, Vodicka E, Clark A, Farewar F, Zhwak ZA, et al. Potential health impact and cost-effectiveness of bivalent human papillomavirus (HPV) vaccination in Afghanistan. Vaccine. 2020;38(6):1352-62. Epub 2019/12/25. doi: 10.1016/j.vaccine.2019.12.013. PubMed PMID: 31870571; PubMed Central PMCID: PMCPMC6997884.

10. Center for Disease Control and Prevention (CDC). The pink book. Rotavirus. 2015. Available from: <<https://www.cdc.gov/vaccines/pubs/pinkbook/rota.html>>.
