# Supplementary material for: Evaluation of rotavirus, pneumococcal conjugate and human papillomavirus vaccination in four Pacific island countries: A cost-effectiveness modelling study
Source: PLoS Med. 2026 Feb 12;23(2):e1004604. doi: 10.1371/journal.pmed.1004604 (PMC12900362; doi:10.1371/journal.pmed.1004604)
Supplement: S2 Appendix — (DOCX) [file pmed.1004604.s005.docx]

**S2 Appendix**

**Input parameters for estimating costs**

**Table A: Inputs for vaccine program costs.** All inputs are based on UNICEF estimates, which were revised to reflect the unique settings (small island nations) in consultation with program managers involved in vaccine programs in these countries.

|  | **Base Case** | **Low** | **High** |
| --- | --- | --- | --- |
| **Supplies^a^ (per dose)** |  |  |  |
| Syringe | $0·05 | $0·04 | $0·06 |
| Safety box/bag | $0·01 | $0·01 | $0·01 |
| **Wastage^b^** |  |  |  |
| Lower price RVV (Tuvalu) | 80% | 50% | 80% |
| Lower price RVV (all other countries) | 50% | 25% | 50% |
| All other vaccines | 10% | 5% | 10% |
| Syringes | 10% | 5% | 10% |
| Safety boxes/bags | 10% | 5% | 10% |
| **Other charges (% of vaccine price)** |  |  |  |
| International handling (all vaccines) | 4% | 4% | 4% |
| International delivery (PCV/HPVV) | 15% | 11% | 19% |
| International delivery (RVV) | 14% | 10% | 18% |

HPVV: Human papillomavirus vaccine; PCV: Pneumococcal conjugate vaccine; RVV: Rotavirus vaccine

^a^ These costs only apply to HPVV and PCV as RVV vaccine does not require syringes.

^b^ Does not consider the additional reserve stock in the base case analysis

**Table B: Inputs for government healthcare costs. All costs scaled to 2019 USD using country-specific GDP deflators.**

|  | **Samoa** | **Tonga** | **Tuvalu** | **Vanuatu** | **Source/notes** |
| --- | --- | --- | --- | --- | --- |
| **Outpatient visit** |  |  |  |  |  |
| Unit cost at hospital (high) | $11·77 | $10·84 | $11·25 | $9·70 | VCH Costing Study [1]^a^ |
| Unit cost at hospital (base case) | $8·59 | $7·91 | $8·20 | $7·08 | WHO-CHOICE^b^ |
| Unit cost at hospital (low) | $6·44 | $5·93 | $6·15 | $5·31 | 75% of base case |
| Cost at lower level facilities relative to hospital | 85% | 85% | 85% | 85% | Based on ratios of WHO-CHOICE costs |
| % visits to hospital vs. lower level facility | 50% | 50% | 61% | 50% | Assumption based on country data^c^ |
| Amoxicillin, 2019 USD | 0.50 | 0.58 | 0.69 | 0.50 | See note^d^ |
| **Cervical cancer hospitalisation** |  |  |  |  |  |
| Unit cost (high) | $89·23 | $81·72 | $71·53 | $72·48 | VCH Costing Study [1]^e^ |
| Unit cost (base case) | $43·20 | $39·56 | $34·63 | $35·09 | WHO-CHOICE^b^ |
| Unit cost (low) | $35·09 | $32·14 | $28·13 | $28·51 | Tonga Report 2007[2] |
| Cost of biopsy | $19·87 | $19·87 | $243·09 | $19·87 | Tonga Report 2007[2] and Tonga new lab charges [3]; Tuvalu lab test costs collected from hospital administrators during field visit. |
| Cost of exam under anesthesia | $154·85 | $154·85 | $154·85 | $154·85 | Tonga Report 2007[2] |
| Total cost abdominal hysterectomy | $340·34 | $311·68 | $272·82 | $276·45 | Tonga Report 2007[2] |
| Average LOS with hysterectomy | 14 days | 14 days | 14 days | 14 days | Tonga Report 2007[2] |
| Average LOS without hysterectomy (low) | 10 days | 10 days | 10 days | 10 days | Tonga Report 2007[2] |
| Average LOS without hysterectomy (base case) | 17 days | 17 days | 17 days | 17 days | Tonga Report 2007[2] |
| Unit cost palliative care (base case) | $44·21 | $44·21 | $44·21 | $44·21 | Campos 2016[4]^f^ |
| Unit cost palliative care (high) | $285·90 | $285·90 | $285·90 | $285·90 | Campos 2016[4]^f^ |
| **Overseas treatment** |  |  |  |  |  |
| % patients referred for overseas treatment |  |  |  |  |  |
| *Local cancer* | 100% | 25% | 100% | 0% | Expert opinion^g^ |
| *Regional cancer* | 5% | 0% | 100% | 0% |  |
| *Distant cancer* | 0% | 0% | 0% | 0% |  |
| Overseas treatment cost | $16,359 | $23,539 | $25,861 | n/a | Country-specific and Blakely 2015[5]^h^ |
| **Paediatric hospitalisation** |  |  |  |  |  |
| Unit cost per bed day (high) | $153·62 | $140·69 | $123·15 | $124·77 | VCH Costing Study [1]^e^ |
| Unit cost per bed day (base case) | $43·2 | $39·56 | $34·63 | $35·09 | WHO-CHOICE^b^ |
| Unit cost per bed day (low) | $35·09 | $32·14 | $28·13 | $28·51 | Tonga Report 2007[2] |
| Ratio of ICU bed day unit cost to regular day | 2 | 2 | 2 | 2 | Expert opinion^g^ |
| % hospitalisations in tertiary hospital vs. lower level facilities | 90% | 80% | 100% | 90% | Assumptions based on limited country data^i^ |
| % severe RVGE hospitalisations to ICU | 20% | 20% | 20% | 20% | Jenney et al. 2009 [6] |
| Average LOS, severe pneumonia | 5 days | 4·5 days | 6·2 days | 4·5 days | Country-specific hospital data^j^ |
| Average LOS, meningitis | 16·3 days | 14·0 days | 14·0 days | 11·6 days | Country-specific hospital data^j^ |
| Average LOS, severe diarrhea | 4·2 days | 4 days | 3·28 days | 4·23 days | Country-specific hospital data^j^ |
| Average LOS, intussusception | 9 days | 9 days | 9 days | 9 days | Assumption |
| Cost of major surgery (base case) | $963·85 | $882·74 | $772·67 | $782·67 | VCH Costing Study [1] and WHO-CHOICE^k^ |
| Cost of major surgery (low) | $269·89 | $247·17 | $216·35 | $219·20 | Tonga Report 2007 [7] and WHO-CHOICE^k^ |

GDP = gross domestic product; ICU: intensive care unit; LOS: length of stay; RVGE: Rotavirus gastroenteritis; VCH: Vila Central Hospital; USD = United States Dollars.

^a^ Costs based on 2011 Vila Central Hospital (VCH) Costing Study from Vanuatu [1]. Costs adjusted to other countries based on ratios with WHO-CHOICE unit cost estimates. Outpatient cost from outpatient department cost per visit.

^b^ WHO-CHOICE unit cost estimates (2011) based on highest level facility.

^c^ For Samoa, Tonga and Vanuatu, based on data from 2016 Annual Report (Vanuatu) [8]. For Tuvalu, based on proportion of urban population.

^d^ For Tonga and Tuvalu, costs based on pharmacy prices collected during field visit. For Samoa and Vanuatu, cost of 0.50 USD assumed.

^e^ Samoa, Tonga and Tuvalu estimates based on Vanuatu estimate, scaled by WHO-CHOICE hospital cost ratio for both countries; Vanuatu estimate from 2011 Vila Central Hospital (VCH) Costing Study [1] cost per bed day in internal medicine (cervical cancer) or paediatric ward (pneumococcal-related diseases and RVGE). Estimates in table represent unit cost in tertiary level hospital.

^f^ Estimates based on Campos et al.[4]. Base case based on lowest cost of palliative care across all countries with primary data available (Campos Table 15), and high estimate based on median cost palliative care across all countries with primary data available. Costs scaled up to 2019 USD using country-specific GDP deflator from original study.

^g^ Based on discussion with hospital administrators in each country

^h^ Samoa estimate based on average cost per patient in 2012 given total number of patients and combined cost of treatment cited in Samoan National Cervical Prevention and Control Policy [9]; Tonga estimate based on cost of treatment in New Zealand [5]; Tuvalu estimate based on mission report [10], conservatively assume this cost includes transportation/ accommodation and per diems.

^i^ For Samoa, based on average across countries; Tonga based on bed occupancy rate in main hospital compared to Vava’u and Ha’apai hospitals; Tuvalu: only 1 hospital in the country; Vanuatu based on data from 2016 Annual Report [8].

^j^ Reported to study team by hospital administrators based on review of hospital records. Samoa: The Tupua Tamases Meaole Hospital; Tonga: Tonga Hospital; Tuvalu: Princess Margaret Hospital (data from 2014-2016); Vanuatu: VCH Paediatrics (data from 2016-2017) For Tonga and Tuvalu, average length of stay for meningitis estimated as average of Samoa and Vanuatu, given lack of country-specific data in these cases.

^k^ Used for cost of surgery related with intussusception. Base case from estimated cost of major survey (2-hour procedure) in Vanuatu VCH Costing Study 2011[1]. Min estimate from Tonga Cervical Cancer 2007 [2]. Report based on cost of major hysterectomy. Base case and min estimates scaled to all other countries using ratio of bed day unit costs from WHO-CHOICE. Costs scaled up to 2019 USD using country-specific GDP deflator from original study.

**Detailed methods for calculating government healthcare costs**

*Outpatient visits*

For acute otitis media and non-severe pneumonia, total outpatient visit costs were based on unit costs (Table B) with the addition of treatment with amoxicillin (dosage from Temple et al 2012[11] and unit cost from Tonga and Tuvalu pharmacies). Cost of outpatient visits for severe pneumonia/meningitis, NPNM, and meningitis sequelae was assumed to be twice the cost of non-severe pneumonia. For RVGE, total outpatient visit costs were based on unit costs (Table B) with the addition of three days of oral rehydration salts (three packets per day), where costs were based on Tuvalu pharmacy prices collected in country. The cost of outpatient visit for severe diarrhea was assumed same as non-severe (assuming all severe cases end up hospitalized).

Unit costs per visit were multiplied by the number of visits per patient based on assumed rates of care-seeking. We assumed one health care visit per two cases of acute otitis media. Health care visits for non-severe pneumonia and non-severe RVGE were based on country-specific care-seeking behaviour for acute respiratory infection in children under 5 years from Demographic and Health Survey (DHS) reports [12-15]. For Tonga and Tuvalu, where care-seeking behaviour for acute respiratory infection was not available in survey reports, estimates were based on care seeking for children with fever (Tonga) and diarrhoea (Tuvalu). We assume 100% of severe pneumonia incur one healthcare visit.

*Hospitalisation*

The total cost of hospitalisation for local cancer was calculated to include the cost of two hospital visits, diagnostic tests (biopsy and exam under anaesthesia), and country specific treatment costs. For Samoa and Tuvalu, we assume 0% hysterectomy prior to overseas transfer. For Tonga, we assume 100% hysterectomy in addition to airfare costs to New Zealand ($450). For Vanuatu we assume 50% hysterectomy. For Samoa, Tuvalu and Vanuatu, the sensitivity analysis used a low estimate of 0.5 × the base case, and a high estimate of 1.5 × the base case. For Tonga, the low estimate uses lower inpatient unit cost estimate from Tonga Cervical Cancer Report 2007 [2] and assumes no exam under anaesthesia, 10-day LOS, and 10% overseas referrals. The high estimate uses Tonga Hospital cost estimate per bed day costs.

The total cost of hospitalisation for regional cancer was calculated to include the cost of two hospital visits, diagnostic tests (biopsy and exam under anaesthesia), and country specific treatment costs. For Samoa and Tonga we assume 25% hysterectomy and 100% palliative care among those ineligible for overseas treatment. For Tuvalu, we assume 0% hysterectomy. For Vanuatu, we assume 50% hysterectomy and 100% palliative care. For Samoa, Tuvalu and Vanuatu, the sensitivity analysis used a low estimate of 0.5 × the base case, and a high estimate of 1.5 × the base case. For Tonga, the low estimate uses lower inpatient unit cost estimate from Tonga Cervical Cancer Report 2007[2] and assumes no exam under anaesthesia, 10 day LOS, and 10% overseas referrals. The high estimate uses Tonga Hospital cost estimate per bed day and high cost of palliative care [4].

The total cost of hospitalisation for distant cancer was calculated to include the cost of two hospital visits, diagnostic tests (biopsy and exam under anaesthesia) and 100% palliative care. For the sensitivity analysis, the low estimate uses the minimum length of stay for no hysterectomy (10 days), assumes no exam under anaesthesia, and for Tonga, uses low inpatient unit cost from Tonga Cervical Cancer Report 2007 [2]. For Tuvalu, the low estimate assumes 10% overseas referral rate. The high estimates use high palliative care costs from Campos et al. 2016 [4] and high unit bed day costs. For Tuvalu, the high estimate assumes 75% overseas referral rate.

For severe pneumonia, meningitis and NPNM, the total hospitalisation costs were calculated as disease and country specific length of stay multiplied by the cost per bed day (accounting for proportion of inpatient stays at lower level facility versus tertiary level hospital) with the addition of the cost of amoxicillin. For Tonga and Tuvalu, we assume 100% blood cultures performed at a cost of $14 in Tonga (based on Tonga Lab Fee schedule, collected from hospital administrators during field visit) and $50 in Tuvalu (based on Tuvalu Lab prices, collected from hospital administrators during field visit). For sensitivity analysis, the low estimate uses unit costs for bed days from the Tonga Cervical Cancer 2007 Report [2]. The high estimate uses unit costs for bed days from Vanuatu VCH Costing Study 2011 [1].

The cost of hospitalisation for RVGE was calculated as length of stay for severe diarrhea multiplied by the cost per bed day (accounting for proportion of inpatient stays at lower level facility versus tertiary level hospital) with the addition of the cost of ICU stay and the cost of drugs (unit drug costs from Tuvalu and Tonga Pharmacies). In Samoa, we assume IV fluids and zinc are provided. In Tuvalu, we assume oral rehydration salts, zinc, IV fluids are provided and antibiotics in 25% of cases. In Tonga and Vanuatu we assume IV fluids are provided. For Tuvalu, costs also assume 100% full blood count ($4·28) + 25% blood cultures ($49·55) and 25% stool test ($46·78) performed (unit costs based on Tuvalu Lab prices, collected from hospital administrators during field visit). For sensitivity analysis, the low estimate value uses Tonga Cervical Cancer 2007 [2] Report for inpatient day costs, while the high estimate uses Vanuatu VCH Costing Study 2011 inpatient day costs [1].

For intussusception, hospitalisation costs were calculated as length of stay multiplied by cost per bed day (assuming all inpatient stays at tertiary level hospital) with the addition of the cost of major surgery. For sensitivity analysis, the low estimate used Tonga Cervical Cancer 2007 Report for inpatient day and cost of major surgery. In Tuvalu, the low estimate assumes no surgery. The high estimate uses Vanuatu VCH Costing Study 2011 for inpatient bed day costs [1].

Unit costs per hospitalisation were multiplied by the number of hospitalisations per patient based on assumed rates of care-seeking. We assume 80-90% of severe pneumonia are hospitalised, based on underlying child mortality and geographic access to hospitals. We assume 100% of meningitis, NPNM, intussusception and severe RVGE are hospitalised. For cervical cancer, hospital utilisation patterns for early-stage cancer were based on country-specific pap smear data, assuming a proportion of women who receive 1 pap smear in 10 years as proxy.

# References

1. Keane C. Hospital Costing Study: Port Vila Central Hospital. Canberra: AusAID, Health Resource Facility, 2011.

2. Russell F. Report on the Burden of Cervical Cancer and Cost Benefit Analysis of HPV Vaccine and Evaluation of Hib Disease Post Hib Vaccine Introduction in The Kingdom of Tonga. Melbourne, Australia: University of Melbourne, 2007.

3. Kingdom of Tonga: Health Services (Fees and Charges) Regulations, (2017).

4. Campos NG, Sharma M, Clark A, Lee K, Geng F, Regan C, et al. Comprehensive Global Cervical Cancer Prevention. Costs and Benefits of Scaling up within a Decade. Boston, Massachusetts Center for Health Decision SCience, Harvard School of Public Health, 2016.

5. Blakely T, Atkinson J, Kvizhinadze G, Nghiem N, McLeod H, Davies A, Wilson N. Updated New Zealand health system cost estimates from health events by sex, age and proximity to death: further improvements in the age of 'big data'. N Z Med J. 2015;128(1422):13-23. Epub 20150925. PubMed PMID: 26411843.

6. Jenney A, Tikoduadua L, Buadromo E, Barnes G, Kirkwood CD, Boniface K, et al. The burden of hospitalised rotavirus infections in Fiji. Vaccine. 2009;27 Suppl 5:F108-11. Epub 2009/11/26. doi: 10.1016/j.vaccine.2009.08.071. PubMed PMID: 19931707.

7. Russell F. Report on Burden of Cervical Cancer and Cost Benefit Analysis of HPV Vaccine and Evaluation of Hib Disease Post Hib Vaccine Introduction in the Kingdom of Tonga. . 2007.

8. Vila Central Hospital Health Information System Officer. Health Information System Annual Report. Port Vila, Vanuatu: 2016.

9. Ministry of Helath. National Cervical Prevention and Control Policy. Government of Samoa, 2014 25 March 2014. Report No.

10. Russell F. Mission Report: Tuvalu. Melbourne, Australia: Prepared for Asian Development Bank, 2017.

11. Temple B, Griffiths UK, Mulholland EK, Ratu FT, Tikoduadua L, Russell FM. The cost of outpatient pneumonia in children <5 years of age in Fiji. Trop Med Int Health. 2012;17(2):197-203. Epub 2011/10/20. doi: 10.1111/j.1365-3156.2011.02897.x. PubMed PMID: 22008519.

12. Central Statistics Division, the Secretariat of the Pacific Community, Macro International Inc. Tuvalu Demographic and Health Survey. Noumea, New Caledonia: 2007.

13. Tonga Ministry of Health, Tonga Department of Statistics, the Secretariat of the Pacific Community, United Nations Population Fund. Tonga Demographic and Health Survey, 2012. Noumea, New Caledonia: 2013.

14. Vanuatu Ministry of Health, Vanuatu National Statistics Office, the Secretariat of the Pacific Community. Vanuatu Demographic and Health Survey 2013. Noumea, New Caledonia: 2014.

15. Census-Surveys and Demography Division. Samoa Demographic and Health Survey 2014. Apia, Samoa: Samoa Bureau of Statistics, Government of Samoa, 2015.
