# Supplementary material for: Evaluation of rotavirus, pneumococcal conjugate and human papillomavirus vaccination in four Pacific island countries: A cost-effectiveness modelling study
Source: PLoS Med. 2026 Feb 12;23(2):e1004604. doi: 10.1371/journal.pmed.1004604 (PMC12900362; doi:10.1371/journal.pmed.1004604)
Supplement: S3 Appendix — (DOCX) [file pmed.1004604.s006.docx]

**S3 Appendix**

**Results for cost-effectiveness analysis of lower price vaccines**

**Table A: Base-case cost-effectiveness results for lower price vaccines.** Incremental cost-effectiveness ratios for each vaccine and all three vaccines denote cost per DALY averted compared to the status quo (10-year program, lifetime benefit stream).

|  | **Samoa** | **Tonga** | **Tuvalu** | **Vanuatu** |
| --- | --- | --- | --- | --- |
| **PCV** |  |  |  |  |
| Vaccine program costs (USD) | 826,791 | 928,886 | 149,116 | 2,196,418 |
| Healthcare cost savings (USD) | 124,893 | 66,627 | 7,764 | 204,474 |
| Outpatient visits averted | 1,878 | 1,087 | 106 | 3,672 |
| Hospitalisations averted | 488 | 312 | 28 | 1204 |
| Deaths averted | 23 | 16 | 2 | 60 |
| DALYs averted | 622 | 421 | 56 | 1582 |
| ICER (USD/DALY averted) | 1,128 | 2,050 | 2,517 | 1,259 |
| **RVV** |  |  |  |  |
| Vaccine program costs (USD) | 522,318 | 742,635 | 158,601 | 1,677,171 |
| Healthcare cost savings (USD) | 175,123 | 105,652 | 8,048 | 149,879 |
| Outpatient visits averted | 11,216 | 5,478 | 526 | 11,125 |
| Hospitalisations averted | 524 | 444 | 32 | 561 |
| Deaths averted | 11 | 7 | 1 | 11 |
| DALYs averted | 310 | 184 | 19 | 309 |
| ICER (USD/DALY averted) | 1,119 | 3,456 | 7,926 | 4,947 |
| **HPVV** |  |  |  |  |
| Vaccine program costs (USD) | 254,278 | 261,233 | 32,937 | 525,870 |
| Healthcare cost savings (USD) | 20,615 | 6,171 | 9,849 | 31,803 |
| Hospitalisations averted | 43 | 22 | 2 | 129 |
| Deaths averted | 219 | 124 | 13 | 530 |
| DALYs averted | 1036 | 556 | 58 | 2267 |
| CER (USD/DALY averted) | 226 | 378 | 401 | 218 |
| **All three vaccines** |  |  |  |  |
| ICER (USD/DALY averted) | 652 | 1,472 | 2,368 | 965 |
| ICER as a percentage of GDP (2019) | 15% | 34% | 58% | 32% |
| Societal ICER (USD/DALY averted) | 621 | 1,440 | 2,346 | 928 |
| Societal ICER as a percentage of GDP (2019) | 14% | 33% | 58% | 30% |

ICER: Incremental cost-effectiveness ratio; DALY: Disability-adjusted life year; GDP: Gross domestic product; HPVV: Human papillomavirus vaccine; PCV: Pneumococcal conjugate vaccine; RVV: Rotavirus vaccine; USD: United States Dollars

**Fig A: Costs and DALY averted results from probabilistic sensitivity analysis of each lower price vaccine and the combined program in each country.**

**
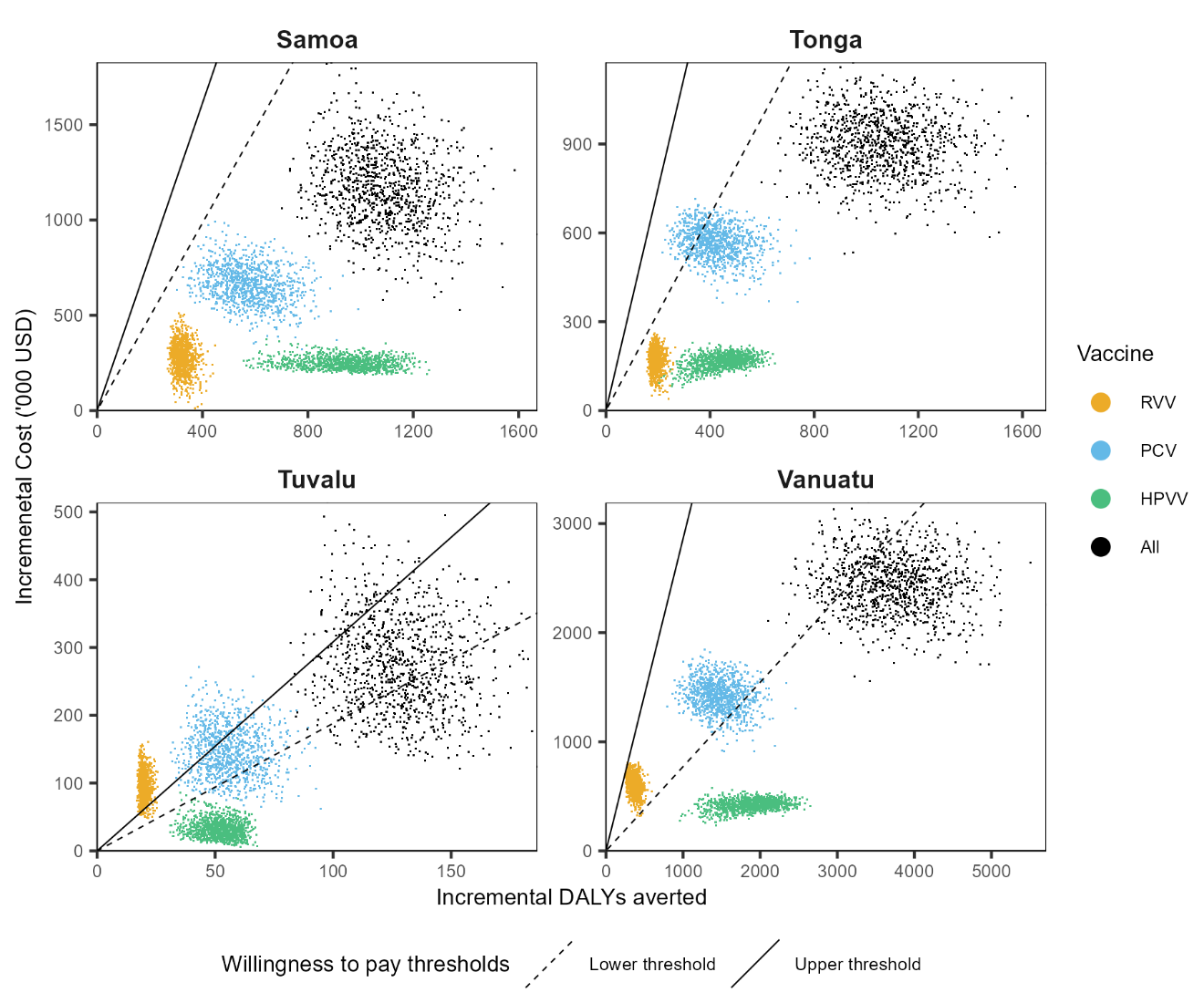
**

Upper WTP estimate is 1× GDP per capita in each country. Dashed black line: Lower WTP estimate is 0·61×, 0·44×, 0·61×, and 0·27× GDP per capital in Samoa, Tonga, Tuvalu and Vanuatu, respectively. DALY: Disability-adjusted life year; HPVV: Human papillomavirus vaccine; PCV: Pneumococcal conjugate vaccine; RVV: Rotavirus vaccine; USD: United States Dollars; WTP: Willingness to pay

**Fig B: One-way sensitivity analysis of the ICERs for lower price vaccines** **in each country.**

**
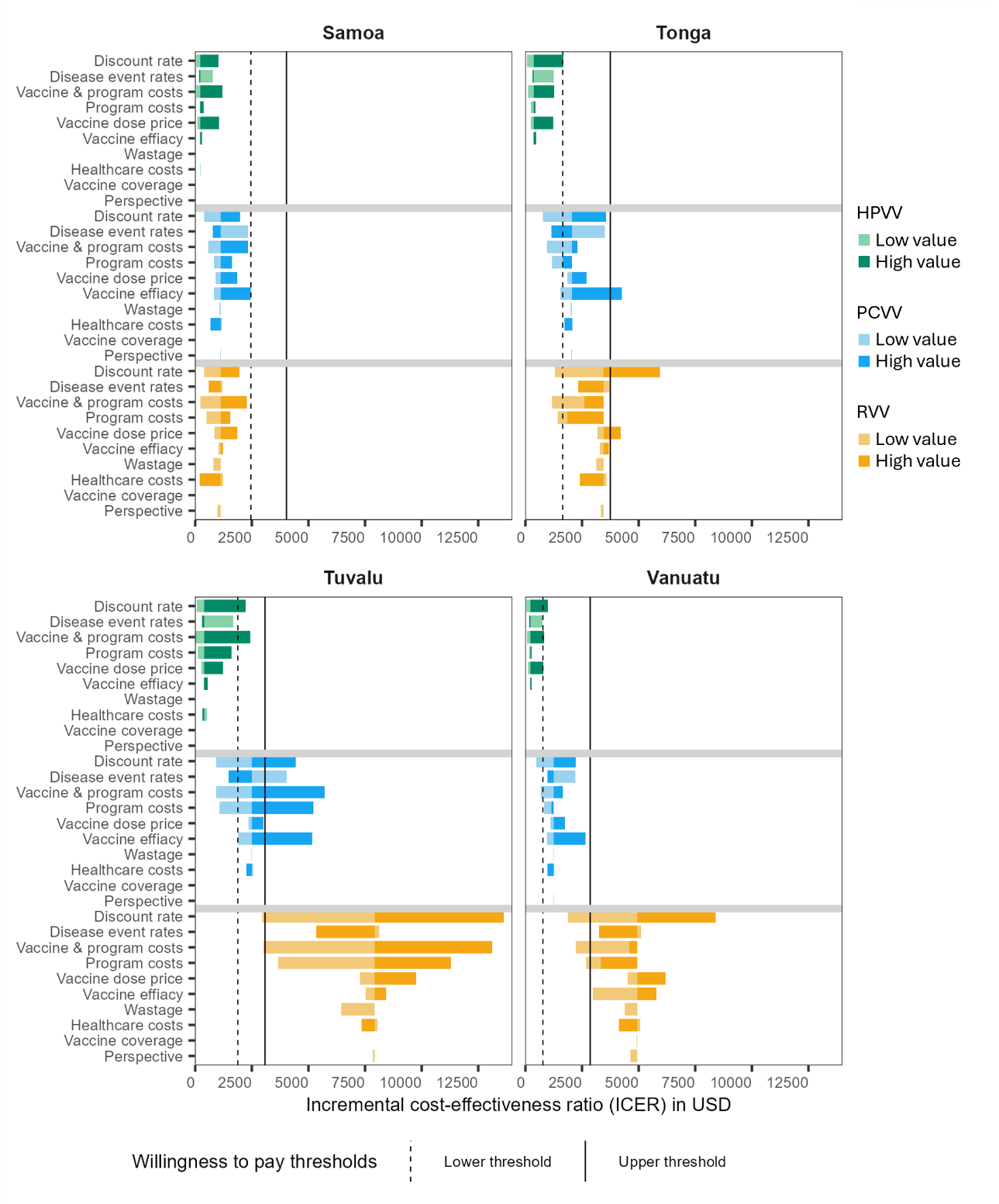
**

DALY: Disability-adjusted life year; PCV: Pneumococcal conjugate vaccine; RVV: Rotavirus vaccine; USD: United States Dollars; WTP: willingness to pay

Disease event rates include upper and lower estimates for both incidence and mortality. Vaccine efficacy includes upper and lower estimates for both efficacy and duration of effect. Upper and lower estimates of vaccine dose price represent the range of quoted prices from different suppliers of the same vaccine. Upper WTP estimate is 1× GDP per capita in each country. Lower WTP estimate is 0·61×, 0·44×, 0·61×, and 0·27× GDP per capital in Samoa, Tonga, Tuvalu and Vanuatu, respectively.

**Fig C: Cost-effectiveness acceptability curves for each lower price vaccine and the combined program in each country.**

**
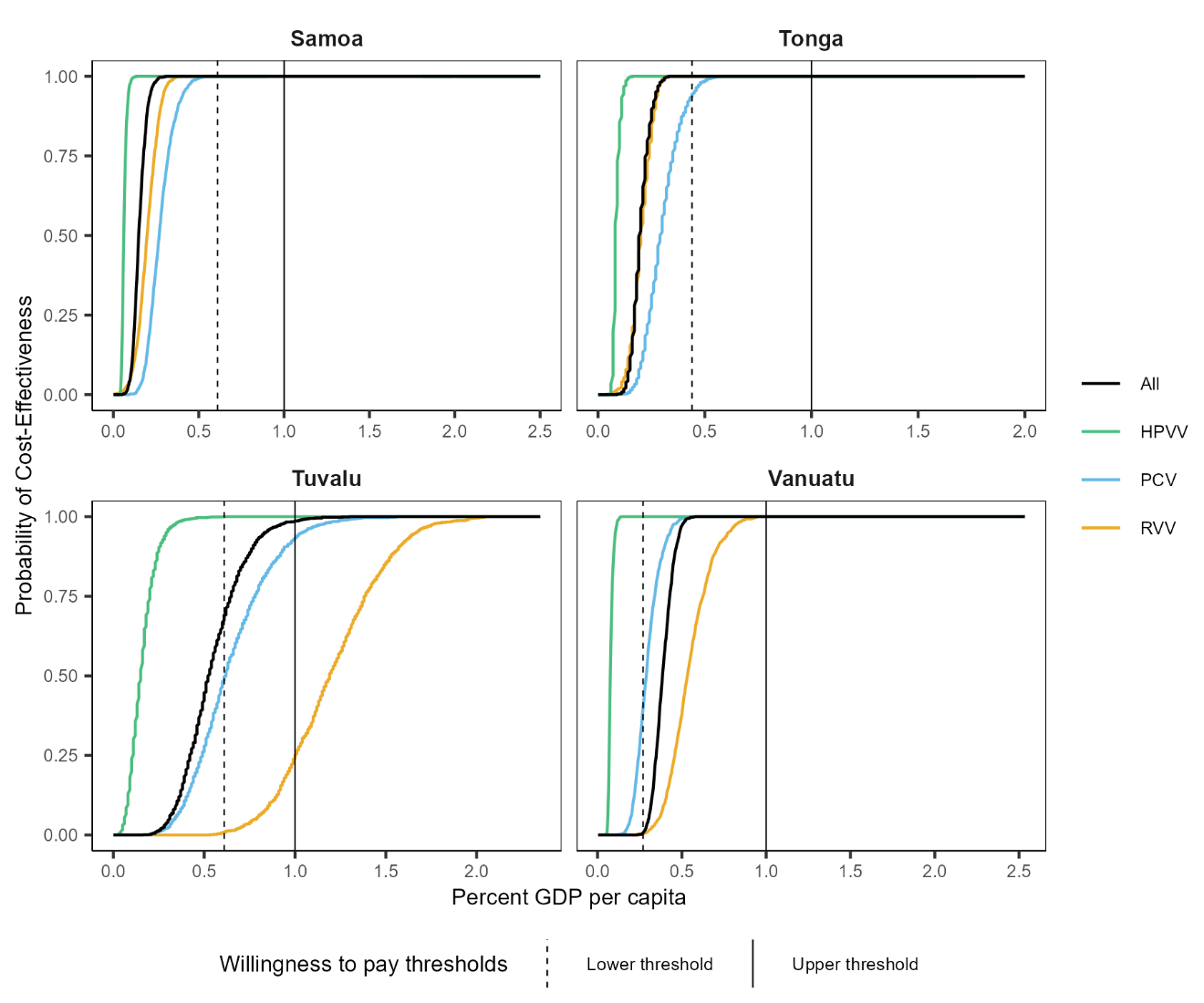
**

GDP: Gross domestic product; HPVV: Human papillomavirus vaccine; PCV: Pneumococcal conjugate vaccine; RVV: Rotavirus vaccine; WTP: Willingness to pay

Vertical lines represent WTP thresholds as a percentage of per capita GDP. Upper WTP estimate is 1× GDP per capita in each country. Lower WTP estimate is 0·61×, 0·44×, 0·61×, and 0·27× GDP per capital in Samoa, Tonga, Tuvalu and Vanuatu, respectively

**Fig D: Results from budget impact analysis for lower price vaccines each country.** Underlying budget impact data can be found in Table C.

**

**

HPVV: Human papillomavirus vaccine; PCV: Pneumococcal conjugate vaccine; RVV: Rotavirus vaccine; USD: United States Dollars

Table B: Annual budget impact (‘000 USD) in each country per vaccine at PAHO Revolving Drug Fund pricing for government and donors. Negative values indicate cost savings

|  | Government costs | | | | | | | Donor costs |
| --- | --- | --- | --- | --- | --- | --- | --- | --- |
|  | HPVV | | PCV | | RVV | | All vaccines | All vaccines |
| Year | Program costs | Healthcare costs | Program costs | Healthcare costs | Program costs | Healthcare costs |  |  |
| Samoa |  |  |  |  |  |  |  |  |
| 2021 | $3.03 | -$8.07 | $13.93 | -$14.46 | $8.54 | -$19.16 | -$16.19 | $411.43 |
| 2022 | $9.35 | -$7.98 | $67.95 | -$14.41 | $26.07 | -$19.16 | $61.83 | $334.84 |
| 2023 | $15.52 | -$7.89 | $122.15 | -$14.34 | $43.64 | -$19.15 | $139.92 | $257.22 |
| 2024 | $21.49 | -$7.76 | $176.45 | -$14.34 | $61.24 | -$19.22 | $217.86 | $179.36 |
| 2025 | $27.28 | -$7.63 | $230.93 | -$14.37 | $78.90 | -$19.34 | $295.77 | $101.50 |
| 2026 | $35.49 | -$7.50 | $297.51 | -$14.32 | $105.10 | -$19.34 | $396.93 | $0.00 |
| 2027 | $34.97 | -$7.37 | $297.45 | -$14.32 | $105.07 | -$19.40 | $396.40 | $0.00 |
| 2028 | $34.45 | -$7.24 | $297.57 | -$14.32 | $105.12 | -$19.48 | $396.11 | $0.00 |
| 2029 | $34.39 | -$7.25 | $298.34 | -$14.37 | $105.39 | -$19.61 | $396.90 | $0.00 |
| 2030 | $34.33 | -$7.25 | $300.06 | -$14.44 | $106.00 | -$19.77 | $398.94 | $0.00 |
| Tonga |  |  |  |  |  |  |  |  |
| 2021 | $6.27 | -$2.38 | $31.18 | -$7.55 | $20.14 | -$11.21 | $36.46 | $280.65 |
| 2022 | $9.60 | -$2.35 | $63.56 | -$7.59 | $30.30 | -$11.29 | $82.23 | $238.23 |
| 2023 | $12.87 | -$2.33 | $96.84 | -$7.65 | $40.76 | -$11.37 | $129.13 | $195.16 |
| 2024 | $16.06 | -$2.30 | $131.03 | -$7.73 | $51.51 | -$11.49 | $177.09 | $151.06 |
| 2025 | $19.19 | -$2.27 | $165.79 | -$7.83 | $62.41 | -$11.64 | $225.66 | $105.68 |
| 2026 | $28.10 | -$2.24 | $232.89 | -$7.88 | $94.75 | -$11.72 | $333.90 | $0.00 |
| 2027 | $27.82 | -$2.21 | $234.55 | -$7.96 | $95.43 | -$11.83 | $335.81 | $0.00 |
| 2028 | $27.54 | -$2.18 | $235.95 | -$8.03 | $96.00 | -$11.93 | $337.35 | $0.00 |
| 2029 | $27.65 | -$2.20 | $237.26 | -$8.08 | $96.53 | -$12.02 | $339.15 | $0.00 |
| 2030 | $27.77 | -$2.22 | $238.58 | -$8.15 | $97.06 | -$12.11 | $340.93 | $0.00 |
| Tuvalu |  |  |  |  |  |  |  |  |
| 2021 | $1.27 | -$3.79 | $6.15 | -$0.88 | $4.10 | -$0.85 | $5.99 | $34.18 |
| 2022 | $1.61 | -$3.75 | $9.46 | -$0.88 | $5.16 | -$0.86 | $10.74 | $29.92 |
| 2023 | $1.94 | -$3.71 | $12.87 | -$0.89 | $6.26 | -$0.86 | $15.61 | $25.61 |
| 2024 | $2.27 | -$3.67 | $16.36 | -$0.90 | $7.39 | -$0.87 | $20.58 | $21.20 |
| 2025 | $2.59 | -$3.62 | $19.91 | -$0.91 | $8.54 | -$0.89 | $25.62 | $16.64 |
| 2026 | $4.10 | -$3.57 | $29.91 | -$0.92 | $14.03 | -$0.89 | $42.65 | $0.00 |
| 2027 | $4.06 | -$3.53 | $30.12 | -$0.93 | $14.13 | -$0.90 | $42.95 | $0.00 |
| 2028 | $4.02 | -$3.48 | $30.30 | -$0.94 | $14.21 | -$0.91 | $43.20 | $0.00 |
| 2029 | $4.03 | -$3.51 | $30.47 | -$0.94 | $14.29 | -$0.91 | $43.42 | $0.00 |
| 2030 | $4.05 | -$3.55 | $30.64 | -$0.95 | $14.37 | -$0.92 | $43.64 | $0.00 |
| Vanuatu |  |  |  |  |  |  |  |  |
| 2021 | $10.52 | -$10.74 | $68.75 | -$23.50 | $44.62 | -$14.45 | $75.20 | $767.63 |
| 2022 | $18.48 | -$10.79 | $161.10 | -$23.61 | $73.68 | -$14.52 | $204.34 | $638.15 |
| 2023 | $26.52 | -$10.85 | $251.83 | -$23.69 | $102.10 | -$14.57 | $331.35 | $505.86 |
| 2024 | $34.74 | -$10.93 | $341.32 | -$23.78 | $130.13 | -$14.62 | $456.86 | $375.09 |
| 2025 | $43.08 | -$11.00 | $431.59 | -$23.90 | $158.50 | -$14.69 | $583.59 | $246.88 |
| 2026 | $53.93 | -$11.08 | $589.69 | -$24.05 | $231.97 | -$14.79 | $825.68 | $0.00 |
| 2027 | $54.35 | -$11.15 | $594.49 | -$24.22 | $233.86 | -$14.89 | $832.43 | $0.00 |
| 2028 | $54.77 | -$11.23 | $600.89 | -$24.41 | $236.38 | -$15.01 | $841.39 | $0.00 |
| 2029 | $55.10 | -$11.27 | $607.52 | -$24.61 | $238.98 | -$15.13 | $850.59 | $0.00 |
| 2030 | $55.43 | -$11.32 | $613.31 | -$24.81 | $241.26 | -$15.26 | $858.61 | $0.00 |

Table C: Annual budget impact (‘000 USD) in each country per lower price vaccine for government and donors. Negative values indicate cost savings.

|  | Government costs | | | | | | | Donor costs |
| --- | --- | --- | --- | --- | --- | --- | --- | --- |
|  | HPVV | | PCV | | RVV | | All vaccines | All vaccines |
| Year | Program costs | Healthcare costs | Program costs | Healthcare costs | Program costs | Healthcare costs |  |  |
| Samoa |  |  |  |  |  |  |  |  |
| 2021 | $3.03 | -$8.07 | $13.93 | -$14.46 | $12.25 | -$20.02 | -$13.33 | $153.63 |
| 2022 | $7.89 | -$7.98 | $27.48 | -$14.41 | $19.23 | -$20.02 | $12.19 | $128.55 |
| 2023 | $12.62 | -$7.89 | $41.05 | -$14.34 | $26.20 | -$20.01 | $37.63 | $103.20 |
| 2024 | $17.20 | -$7.76 | $54.64 | -$14.34 | $33.18 | -$20.08 | $62.85 | $77.83 |
| 2025 | $21.65 | -$7.63 | $68.28 | -$14.37 | $40.19 | -$20.21 | $87.90 | $52.56 |
| 2026 | $28.55 | -$7.50 | $94.20 | -$14.32 | $59.51 | -$20.21 | $140.22 | $0.00 |
| 2027 | $28.13 | -$7.37 | $94.18 | -$14.32 | $59.50 | -$20.27 | $139.84 | $0.00 |
| 2028 | $27.71 | -$7.24 | $94.22 | -$14.32 | $59.52 | -$20.35 | $139.55 | $0.00 |
| 2029 | $27.67 | -$7.25 | $94.46 | -$14.37 | $59.67 | -$20.49 | $139.70 | $0.00 |
| 2030 | $27.62 | -$7.25 | $95.00 | -$14.44 | $60.02 | -$20.65 | $140.30 | $0.00 |
| Tonga |  |  |  |  |  |  |  |  |
| 2021 | $6.27 | -$2.38 | $31.18 | -$7.55 | $30.20 | -$11.76 | $45.98 | $139.44 |
| 2022 | $8.82 | -$2.35 | $39.56 | -$7.59 | $34.66 | -$11.83 | $61.26 | $126.01 |
| 2023 | $11.32 | -$2.33 | $48.20 | -$7.65 | $39.29 | -$11.92 | $76.92 | $112.50 |
| 2024 | $13.76 | -$2.30 | $57.07 | -$7.73 | $44.04 | -$12.04 | $92.80 | $98.72 |
| 2025 | $16.16 | -$2.27 | $66.04 | -$7.83 | $48.81 | -$12.20 | $108.71 | $84.51 |
| 2026 | $24.35 | -$2.24 | $107.07 | -$7.88 | $85.60 | -$12.29 | $194.60 | $0.00 |
| 2027 | $24.11 | -$2.21 | $107.83 | -$7.96 | $86.21 | -$12.40 | $195.58 | $0.00 |
| 2028 | $23.86 | -$2.18 | $108.47 | -$8.03 | $86.72 | -$12.51 | $196.34 | $0.00 |
| 2029 | $23.96 | -$2.20 | $109.08 | -$8.08 | $87.21 | -$12.60 | $197.36 | $0.00 |
| 2030 | $24.06 | -$2.22 | $109.68 | -$8.15 | $87.69 | -$12.69 | $198.36 | $0.00 |
| Tuvalu |  |  |  |  |  |  |  |  |
| 2021 | $1.27 | -$3.79 | $6.15 | -$0.88 | $6.05 | -$0.90 | $7.91 | $23.84 |
| 2022 | $1.53 | -$3.75 | $7.03 | -$0.88 | $7.16 | -$0.90 | $10.19 | $21.96 |
| 2023 | $1.78 | -$3.71 | $7.95 | -$0.89 | $8.31 | -$0.91 | $12.53 | $20.06 |
| 2024 | $2.03 | -$3.67 | $8.89 | -$0.90 | $9.49 | -$0.92 | $14.92 | $18.13 |
| 2025 | $2.27 | -$3.62 | $9.83 | -$0.91 | $10.67 | -$0.93 | $17.32 | $16.12 |
| 2026 | $3.71 | -$3.57 | $17.19 | -$0.92 | $18.28 | -$0.94 | $33.75 | $0.00 |
| 2027 | $3.67 | -$3.53 | $17.31 | -$0.93 | $18.41 | -$0.94 | $33.99 | $0.00 |
| 2028 | $3.63 | -$3.48 | $17.41 | -$0.94 | $18.52 | -$0.95 | $34.20 | $0.00 |
| 2029 | $3.65 | -$3.51 | $17.51 | -$0.94 | $18.62 | -$0.96 | $34.37 | $0.00 |
| 2030 | $3.66 | -$3.55 | $17.61 | -$0.95 | $18.73 | -$0.97 | $34.54 | $0.00 |
| Vanuatu |  |  |  |  |  |  |  |  |
| 2021 | $12.16 | -$10.74 | $68.75 | -$23.50 | $65.86 | -$16.81 | $95.73 | $352.14 |
| 2022 | $19.02 | -$10.79 | $91.81 | -$23.61 | $77.68 | -$16.90 | $137.22 | $310.48 |
| 2023 | $25.95 | -$10.85 | $114.16 | -$23.69 | $88.95 | -$16.95 | $177.57 | $267.37 |
| 2024 | $33.13 | -$10.93 | $136.19 | -$23.78 | $100.05 | -$17.02 | $217.65 | $224.76 |
| 2025 | $40.46 | -$11.00 | $158.65 | -$23.90 | $111.52 | -$17.10 | $258.63 | $183.23 |
| 2026 | $60.25 | -$11.08 | $247.19 | -$24.05 | $188.75 | -$17.21 | $443.85 | $0.00 |
| 2027 | $60.88 | -$11.15 | $249.20 | -$24.22 | $190.29 | -$17.33 | $447.66 | $0.00 |
| 2028 | $61.51 | -$11.23 | $251.88 | -$24.41 | $192.34 | -$17.47 | $452.62 | $0.00 |
| 2029 | $62.00 | -$11.27 | $254.66 | -$24.61 | $194.46 | -$17.61 | $457.63 | $0.00 |
| 2030 | $62.50 | -$11.32 | $257.09 | -$24.81 | $196.31 | -$17.76 | $462.01 | $0.00 |
